# Mesenchymal stem cells reverse EMT process through blocking the activation of NF-κB and Hedgehog pathways in LPS-induced acute lung injury

**DOI:** 10.1038/s41419-020-03034-3

**Published:** 2020-10-15

**Authors:** Kun Xiao, Wanxue He, Wei Guan, Fei Hou, Peng Yan, Jianqiao Xu, Ting Zhou, Yuhong Liu, Lixin Xie

**Affiliations:** 1 Center of Pulmonary & Critical Care Medicine, Chinese People’s Liberation Army (PLA) General Hospital, Beijing, 100853 China; 2 Medical School of Chinese People’s Liberation Army (PLA), Beijing, 100853 China

**Keywords:** Biotechnology, Cell biology

## Abstract

Acute lung injury (ALI) is a pulmonary disorder, which can result in fibrosis of the lung tissues. Recently, mesenchymal stem cell (MSC) has become a novel therapeutic method for ALI. However, the potential mechanism by which MSC regulates the progression of ALI remains blurry. The present study focused on investigating the mechanism underneath MSC-reversed lung injury and fibrosis. At first, we determined that coculture with MSC led to the inactivation of NF-κB signaling and therefore suppressed hedgehog pathway in LPS-treated MLE-12 cells. Besides, we confirmed that MSC-exosomes were responsible for the inhibition of EMT process in LPS-treated MLE-12 cells through transmitting miRNAs. Mechanism investigation revealed that MSC-exosome transmitted miR-182-5p and miR-23a-3p into LPS-treated MLE-12 cells to, respectively, target Ikbkb and Usp5. Of note, Usp5 interacted with IKKβ to hamper IKKβ ubiquitination. Moreover, co-inhibition of miR-182-5p and miR-23a-3p offset the suppression of MSC on EMT process in LPS-treated MLE-12 cells as well as in LPS-injured lungs of mice. Besides, the retarding effect of MSC on p65 nuclear translocation was also counteracted after co-inhibiting miR-182-5p and miR-23a-3p, both in vitro and in vivo. In summary, MSC-exosome transmitted miR-23a-3p and miR-182-5p reversed the progression of LPS-induced lung injury and fibrosis through inhibiting NF-κB and hedgehog pathways via silencing Ikbkb and destabilizing IKKβ.

## Introduction

Acute lung injury (ALI) is featured as diffuse alveolar damage, which can lead to excessive pulmonary inflammation and apoptosis of alveolar epithelial cells (AECs)^1^. The diagnosis of ALI depends on clinical and radiographic criteria, whereas sometimes inaccurate diagnoses are inevitable^2^. Persistent and repetitive injury can induce tissue and cellular responses that ultimately lead to pulmonary fibrosis^3–6^. Researches have shown that epithelial–mesenchymal transition (EMT) is closely associated with the initiation and progression of fibrosis^7–9^. Hence, understanding the molecular mechanisms involved in EMT process of AECs is of significance in improving the treatment of patients with ALI-induced pulmonary fibrosis.

Lipopolysaccharide (LPS) components are known as important inflammatory inducers, which can result in ALI^10^. LPS-induced ALI animal model has been built to investigate the mechanism and possible therapies of ALI^11^. Here, we established ALI model by treating a murine lung epithelial (MLE) cell line MLE-12 with LPS for further study.

Mesenchymal stem cell (MSC) has been proven to be a novel therapeutic method for lung diseases^12,13^, including ALI and pulmonary fibrosis^14,15^. A growing number of studies have provided evidence to show the importance of MSC in treating LPS-induced ALI^16–19^. However, the underlying molecular mechanism by which MSC alleviates ALI remains to be explored.

Signaling pathways are essential participants in the biological processes of ALI and pulmonary fibrosis. Nuclear factor κ-light-chain-enhancer of activated B cells (NF-κB) pathway is a well-known inflammatory pathway, which has been reported to be implicated in LPS-induced ALI^20,21^. Likewise, Hedgehog signaling pathway is also associated with a range of pathological development of multiple diseases and the high expression of sonic hedgehog signaling molecule (Shh) can promote the activation of hedgehog pathway^22^. In our current study, we explored whether these pathways were involved in LPS-induced lung injury and fibrosis.

Increasing evidence suggested that microRNAs (miRNAs) are crucial regulators in ALI. For example, miR-106a and its target TLR4 formed a negative feedback loop to regulate ALI^23^. MiR-150 negatively regulated AKT3 to attenuate LPS-induced ALI^24^. Exosomes are common membrane-bound nanovesicles that include various biomolecules, such as lipids, proteins, and nucleic acids. Exosomes are derived from donor cells through exocytosis and can be absorbed into target cells, therefore transferring biological signals between cells^25^. Recently, MSC has been reported to treat LPS-induced ALI by transporting miRNAs into LPS-induced cells through exosomes^26,27^. Therefore, it is of importance to unveil the regulatory mechanism of miRNAs released by MSC-exosome in treating LPS-induced ALI and pulmonary fibrosis.

In summary, our study focused on exploring the molecular mechanisms that contributed to the therapeutic effect of MSC on LPS-induced ALI.

## Materials and methods

### Hematoxylin–eosin (HE) staining

As previously described^28^, HE staining was used to observe the morphology of lung tissues extracted from male C57BL/6 mice treated with saline solution or LPS (10 mg/kg; Sigma-Aldrich, Missouri, USA). Briefly, the extracted lung tissues were fixed with neutral formalin (10% dilution) for about 24 h, dewaxed with xylene, and dehydrated in ethanol (concentration is 100%, 90% and 70%, respectively). The sections were then stained with hematoxylin for seven minutes and hydrated by 95% ethanol for five seconds. Subsequently, the sections were soaked in ammonia liquor for 30 s until they turned blue. Afterwards, the sections were stained with eosin for about 1 min, dehydrated in gradient ethanol (concentration is 100%, 95%, 75%, and 50%, respectively) twice (2 min each time), and cleared twice in xylene (5 min each time). After the sections were sealed with neutral balsam, the histopathological changes of the different lung tissues were observed under an optical microscope.

### Ethical statements

The experimental procedures were all conducted with the approval from the Animal Care and Use Committee as well as Ethics Committee of Chinese People’s Liberation Army (PLA) General Hospital.

### Reagents

The kinase inhibitor of NF-κB-1 (KINK-1; 5 μM), IKKβ inhibitor, was procured from MedChemExpress (Monmouth Junction, NJ, USA). The cycloheximide (CHX; 5 µM) was purchased from Sigma-Aldrich (St. Louis, MO, USA) and MG132 (10 Μm) was procured from Selleck Chemicals (Houston, TX, USA). The parthenolide (5 μmol/L), p65 inhibitor was purchased from Tocris Bioscience (Ellisville, Missouri, USA). LPS used for this study was obtained from Sigma-Aldrich (Saint-Louis, Missouri, USA).

### In vitro MSCs isolation

C57BL/6 mice were purchased from the Jackson Laboratory (Bar Harbor, ME, USA) and euthanized to remove the femur and tibia under sterile condition. After washing in DMEM (Gibco, Grand Island, NY, USA), the marrow cells were centrifuged for 3 min, and then cultured with 10% fetal bovine serum (Gibco) and 100 U/mL penicillin and streptomycin (Gibco) in DMEM. Following culture in 5% CO_2_ at 37 °C, MSCs were passaged to the third generation when cells reached 70–80% confluence for use.

### LPS induction in AECs

MLE-12 cell line, the type II AECs, was procured from the Shanghai Institute of Biochemistry and Cell Biology, Chinese Academy of Sciences (Shanghai, China) and maintained in 5% CO_2_ at 37 °C. After reaching 80–90% confluence, cells were treated with 500 ng/mL of LPS (Sigma-Aldrich) for 24 h.

### Coculture of MSCs and LPS-treated MLE-12 cells

MSCs (1 × 10^4^ cells/well) were placed into the 24-well basolateral chamber of transwell system, and LPS-treated MLE-12 cells were placed into the apical chamber. LPS-treated MLE-12 cells were cocultured with MSCs in the coculture system for 24 h, and then MLE-12 cells were isolated for analysis.

### Quantitative real-time polymerase chain reaction (RT-qPCR)

Total RNA extraction was first completed in the presence of TRIzol Reagent (Invitrogen, Carlsbad, CA, USA), as guided by provider. After that, 1 μg of total RNA was used for cDNA synthesis with PrimeScript Reverse Transcriptase Kit (Takara, Kyoto, Japan). PCR reaction was undertaken on ABI Prism 7900HT (Applied Biosystems, Foster City, CA, USA) using SYBR GREEN PCR Master (Applied Biosystems). All results were calculated using the 2^−ΔΔCt^ method through normalizing to GAPDH or U6.

### Western blot

After lysing in RIPA lysis buffer, the collected total protein was separated on sodium dodecyl sulfate polyacrylamide gel electrophoresis (SDS-PAGE) (10%), shifted onto polyvinylidene fluoride membranes, and then blocked by 5% nonfat milk. The membranes were probed all night at 4 °C with the primary antibodies against loading control GAPDH (ab8245, 1/1000; Abcam, Cambridge, MA, USA) and p65 (ab16502, 1/1000; Abcam), Histone H3 (ab1791, 1/1000; Abcam), IKKα (ab32041, 1/10,000; Abcam), IKKβ (ab124957, 1/1000; Abcam), NEMO (ab178872, 1/5000; Abcam), p-IKBα(Ser36) (ab133462, 1/10,000; Abcam), p-IKBβ(Ser23) (Cat# 4921 S, 1/1000; Cell Signaling Technology, Danvers, MA, USA), CD9 (ab92726, 1/2000; Abcam), CD63 (ab134045, 1/1000; Abcam), CD81 (ab109201, 1/1000; Abcam), HSP70 (ab2787, 1/1000; Abcam), E-cadherin (ab76055, 1/1000; Abcam), α-SMA (Cat# 19245S, 1/1000; Cell Signaling Technology), TGF-β1 (ab27969, 1/2000; Abcam), Collagen type I (ab34710, 1/1000; Abcam), Collagen type III (ab7778, 1/5000; Abcam), Usp5 (ab154170, 1/1000; Abcam). Next, membranes were washed in TBS-T, and then incubated for 2 h with horseradish peroxidase-labeled secondary antibodies at room temperature. Electrochemiluminescence luminous liquid was employed for analyzing protein bands as instructed by supplier (Pierce, Rockford, IL, USA). Results were visualized after developing in the dark.

### Transfection

After LPS treatment, MLE-12 cells were reaped at logarithmic growth phase and seeded into 6-well plates (1 × 10^6^ cells/well). After cell confluence had reached 80–90%, cells were transfected with the indicated plasmids for 48 h, in the presence of lipofectamine 3000 kit (Invitrogen). The miR-182-5p mimics, miR-182-5p inhibitor and miR-23a-3p mimics, miR-23a-3p inhibitor were constructed by GenePharma (Shanghai, China) along with their corresponding NCs. To silence Dicer expression, the specific shRNA to Dicer was synthesized by GenePharma. In addition, the full-length cDNA sequences of Usp5, Ikbkb, and p65 were individually inserted into the pcDNA3.1 vectors (Invitrogen) for overexpression.

### Luciferase reporter assay

The MLE-12/LPS cells were seeded in 96-well plates and transfected with the indicated luciferase reporter plasmids to detect the luciferase activities of Notch pathway, Wnt pathway, JAK/STAT3 pathway, NF-κB pathway, PI3K/AKT pathway, NRF2 pathway, Hedgehog pathway, MAPK/JNK pathway and MAPK/ERK pathway, respectively. For gene promoter analyses, cells were co-transfected with indicated transfection plasmids and the pGL3-basic reporter vectors (Promega, Madison, WI, USA) containing Ikbkb promoter, Usp5 promoter or SHH promoter. In addition, the Ikbkb fragment covering miR-182-5p wild-type or mutant binding sites was inserted into pmirGLO luciferase reporter vectors (Promega), and then co-transfected with miR-182-5p mimics or NC mimics into MLE-12/LPS cells and HEK-293T cells (ATCC; Manassas, VA, USA). The pmirGLO vectors which contained Usp5 fragment covering miR-23a-3p wild-type or mutant binding sites, were co-transfected with miR-23a-3p mimics or NC mimics into MLE-12/LPS and HEK-293T cells. At 48 h post transfection, all luciferase activities were examined with a luciferase reporter assay system (Promega).

### Isolation of exosomes

The MSCs were collected at third passage and cultured overnight in serum-free medium until reached 80–90% confluence. The MSC conditioned medium was then centrifuged at 300 × *g* for 10 min, 2000 × *g* for 20 min, and 10000 × *g* for 1 h. After removing cellular debris, the supernatant was centrifuged at 10,000 × *g* for 1 h, filtered through multi-pore membrane (0.22 μm) and centrifuged at 10,000 × *g* for 2 h. Next, the precipitates were cultured with 25 mM of HEPES (pH = 7.4) in serum-free medium, and then centrifuged at 10,000 × *g* to acquire the exosomes.

### Nanoparticle tracking analysis (NTA)

The size of exosomes was determined by NTA using NanoSight LM10 instrument (Malvern Instruments Ltd., Malvern, UK) equipped with Viton sample room and laser (640 nm). Exosomes were re-suspended in phosphate-buffered saline (PBS) and then diluted with Milli-Q by 500 times, followed by injection into sample room using sterile syringe. The granularity value was assessed by the NTA software corresponded to the arithmetic value of all particle sizes analyzed by software.

### Transmission electron microscopy (TEM)

To characterize the MSC-exosome and MSC/sh-Dicer-exosome, 30 μL of exosomes was stained in 30 μL of phosphotungstic acid solution (pH = 6.8). Next, exosomes were analyzed using transmission electron microscopy (TEM).

### Exosome labeling

One micrometre of PKH67 (Sigma-Aldrich) was commercially acquired to label the exosomes in line with the established protocol. The labeled MSC-exosome and MSC/sh-Dicer-exosome were added into MLE-12/LPS cells and cultured for 6 h. DAPI solution (Beyotime, Shanghai, China) was applied to stain cell nuclei. The slides were fluorescently observed under a laser scanning microscope (Carl Zeiss Meditec, Oberkochen, Germany).

### Immunofluorescence staining (IF)

MLE-12/LPS cells were placed on culture slides for 24 h, and then rinsed in PBS. After that, cells were fixed by 4% PFA for 10 min and blocked by 5% bovine serum albumin for 10 min. The primary antibody against p65 and secondary antibody were used for incubation in turn. Following washing in PBS, the slides were subjected DAPI staining and fluorescence detection was performed to observe the fluorescence of p65.

### Flow cytometry

Cell apoptosis was studied by use of flow cytometer (BD Biosciences, Franklin Lakes, NJ, USA) via Annexin V/PI double staining method (Invitrogen). The transfected MLE-12/LPS cells were harvested and mixed in 1× binding buffer with FITC-Annexin V and PI for 15 min in the dark. Apoptotic cells were examined by a flow cytometry.

### Co-immunoprecipitation (Co-IP)

The cell lysates were extracted from the treated MLE-12/LPS cells by use of RIPA lysis buffer, and then cultured overnight with specific antibodies against IKKβ, Usp5 and IgG (negative control) in constant speed at 4 °C. Following mixing with Protein A/G-beads, the antigen-antibody mixture was acquired. After washing thrice in IP lysis buffer, western blot analysis was conducted for the eluted proteins. In addition, the proteins were separated on SDS-PAGE for visualization by silver staining.

### Establishment of LPS-induced ALI mouse model

To establish ALI rat model, C57BL/6 mice (8–10 weeks) were subjected to intraperitoneal injection with 13.5 mg/kg acepromazine and 150 mg/kg chloramines for inducing general anesthesia. Then, a midline incision was made in the anterior region of the neck before preforming tracheotomy. Left and right lungs were separately treated with 50 μL of 1 mg/kg LPS solution using micro-sprayer. Four hours later, MSCs (1 × 10^5^) or indicated exosomes (70 μg) were injected into mice by tail vein, followed by appropriate injection of miR-23a-3p antagomir (5 nM per mouse each time) or miR-182-5p antagomir (5 nM per mouse each time). Two days later, mice were sacrificed and lung tissues were collected for subsequent analysis.

### HE staining

Left lung tissues were extracted from mice and then fixed by 10% neutral formalin and dewaxed in xylene, followed by dehydration in ethanol. The sections were stained in hematoxylin for 7 min and hydrated in 95% ethanol for 5 s, followed by socking with weak ammonia liquor for 10–30 s. After turning blue, sections were stained in eosin for 1 min and dehydrated in ethanol, followed by clearing in xylene twice (5 min each). Sections were subsequently sealed with neutral balsam. Histopathological changes of the lung tissues were analyzed by optical microscope. The degree of ALI in mice was semiquantitatively analyzed via determining the histology score from 0 (no lesion) to 4 (major and extended lesions), and the criteria of each score was defined as follow: alveolar necrosis, vascular congestion, infiltration by neutrophils, and infiltration by macrophages.

### Chromatin immunoprecipitation (ChIP)

ChIP assay was undertaken by EZ ChIP^™^ Chromatin Immunoprecipitation Kit, as instructed by provider (Millipore, Bedford, MA, USA). MLE-12/LPS cells were treated with 4% PFA for 15 min’s cross-link, and then with ultrasonic for shearing DNA into 500-bp of fragments. The 6 h of immunoprecipitation was implemented with anti-p65 antibody and control IgG antibody, following addition of 30 μl of magnetic beads. The collected precipitated chromatin was subjected to RT-qPCR.

### Statistical analyses

Each experiment was conducted in triplicate. All data were exhibited as the mean ± standard deviation. Comparisons between two groups were processed with Student’s *t* test. Differences among multiple groups were analyzed by one-way ANOVA. Statistical analyses were made by using SPSS 19.0 software (IBM, Armonk, NY, USA). The *p* value < 0.05 was taken to indicate the statistical significance.

## Results

### NF-κB and hedgehog pathways are inactivated in LPS-treated MLE-12 cells cocultured with MSC

LPS was used to treat C57BL/6 mice through intratracheal injection for 4 h to construct ALI model. After HE staining, the histological characteristics of lung tissues were observed, and we found that the injury in LPS group was much worse than control group (Fig. S1A). Simultaneously, the ALI cell model was constructed by treating MLE-12 cells with 500 ng/mL of LPS for 24 h. As shown in Fig. S1B, cell apoptosis rate was significantly increased after LPS treatment, indicating a success in LPS-induced in vitro ALI model. Continuous lung injury may result in pulmonary fibrosis, which threatens public health. To investigated whether LPS affected the fibrosis of ALI cell model, we evaluated the changes in the EMT process. At first, IF staining revealed that the intensity of E-cadherin was reduced under LPS treatment, whereas that of Vimentin was enhanced (Fig. S1C). Subsequently, we assessed the levels of EMT markers with RT-qPCR and western blot assay. According to the experimental results, the level of epithelial marker (E-cadherin) was decreased, whereas the levels of mesenchymal markers (α-SMA, TGF-β1, Collagen type I, and Collagen type III) were increased in LPS-treated MLE-12 cells (Fig. S1D, E). These data suggested that LPS-induced injury and EMT progress in MLE-12 cells.

**Fig. 1:**
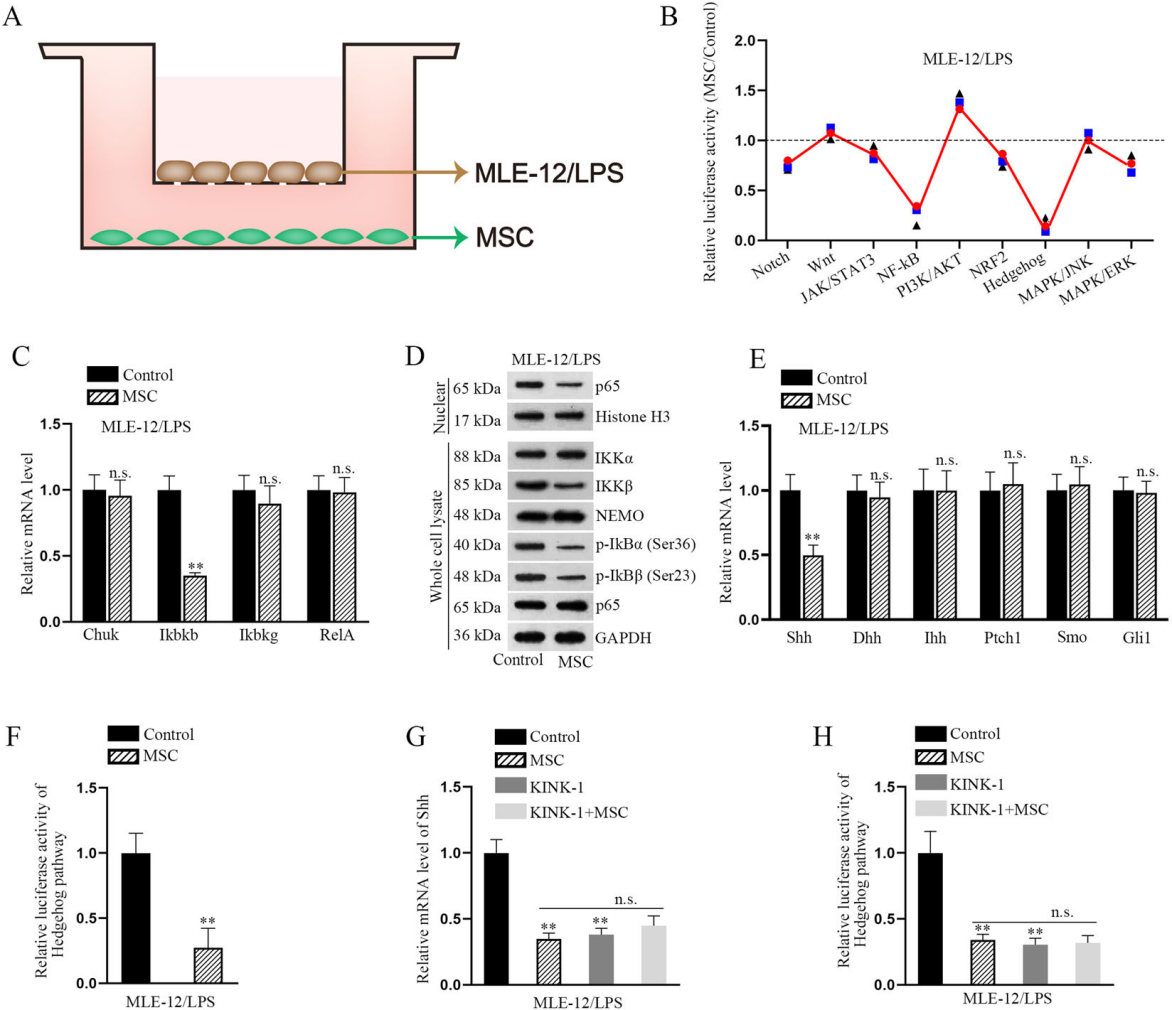
NF-κB and hedgehog signaling pathways are inactivated in LPS-treated MLE-12 cells cocultured with MSC. **a** The cocultured system with LPS-treated MLE-12 cells and MSCs was built. **b** Luciferase reporter assays detected the relative luciferase activity of various signaling pathways (Notch, Wnt, Nanog, NF-κB, PI3K/AKT, Oct4, Hedgehog, MAPK/JNK, and MAPK/ERK) in LPS-treated MLE-12 cells treated with or without MSC. **c** The mRNA level of Ikbkb, Chuk, Ikbkg, or RelA was examined by RT-qPCR after cocultured with MSC. **d** Western blot analysis determined the levels of IKKβ, p-IκBα, and p-IκBβ in the whole cell lysates as well as the nuclear protein level of p65 after cocultured with MSCs. **e** RT-qPCR analyzed the mRNA levels of hedgehog pathway key factors (Shh, Dhh, Ihh, Ptch1, Smo, and Gli1) in control cells and cocultured cells. **f** The luciferase activity of hedgehog pathway was detected in cocultured cells. **g** Relative mRNA level of Shh was measured by RT-qPCR in LPS-treated MLE-12 cells under four different conditions (control, MSC, KINK-1, and KINK-1+MSC). **h** Relative luciferase activity of hedgehog pathway was measured in LPS-treated MLE-12 cells under the same four conditions. ^**^p < 0.01. n.s. no statistical significance.

Recently, studies have shown that MSC contributes to treating LPS-induced ALI^16,18^. We injected the MSC into C57BL/6 mice which had been treated with LPS for the stimulation of in vivo ALI model. Through HE staining, we observed that the lung injury induced by LPS was alleviated after co-treatment with MSC (Fig. S2A). To assess the influence of MSC on LPS-treated MLE-12 cells, the coculture system was constructed (Fig. 1a). In vitro functional assays indicated that cocultured LPS-treated MLE-12 cells presented low apoptosis rate (Fig. S2B). Also, the EMT process was reversed in LPS-treated MLE-12 cells after coculturing with MSC (Fig. S2C–E). Abnormal activation of signaling pathways is closely associated with the course of various human diseases, including ALI and pulmonary fibrosis. In this regard, we measured the activity of some signaling pathways in this coculture system. Intriguingly, we discovered that the activity of NF-κB and hedgehog signaling pathways was significantly weakened in LPS-treated MLE-12 cells after cocultured with MSC (Fig. 1b). To further prove the effect of MSC on the activity of NF-κB and hedgehog pathways, we examined the mRNA and protein levels of some key factors. As a result, the mRNA level of Ikbkb, but not that of Chuk, Ikbkg, or RelA, was attenuated after cocultured with MSC (Fig. 1c). Through western blot analysis, we determined that the levels of nuclear factor IKKβ and its downstream p-IκBα and p-IκBβ in the whole cell lysate as well as the nuclear protein level of p65 was significantly reduced after cocultured with MSC (Fig. 1d). Similarly, the mRNA levels of hedgehog pathway key factors were also evaluated in control cells and cocultured cells. As shown in Fig. 1e, MSC coculture led to a specific decrease of Shh mRNA level, but had no impact on the expression of other factors. The decreased luciferase activity in coculture system further indicated the inactivation of hedgehog pathway in MSC-cocultured cells (Fig. 1f). Previously, Nakashima et al.^29^ have proved that nuclear p65 induces the upregulation of Shh mRNA and contributes to the activation of hedgehog signaling pathway. Therefore, we explored whether the inactivation of hedgehog pathway was caused by the MSC coculture alone or by MSC-inactivated NF-κB pathway. The mRNA level of Shh was measured in LPS-treated MLE-12 cells under four different conditions. As presented in Fig. 1g, h, the mRNA level of Shh and the activity of hedgehog pathway were decreased after treated with MSC or KINK-1 (NF-κB inhibitor). More importantly, the decreased tendency caused by KINK-1 was not changed after co-treatment with MSC. These results indicated that MSC induces the inactivation of hedgehog pathway by weakening the activity of NF-κB pathway.

### Activated NF-κB pathway enhances the activity of hedgehog pathway by transcriptionally activating Shh

**Fig. 2:**
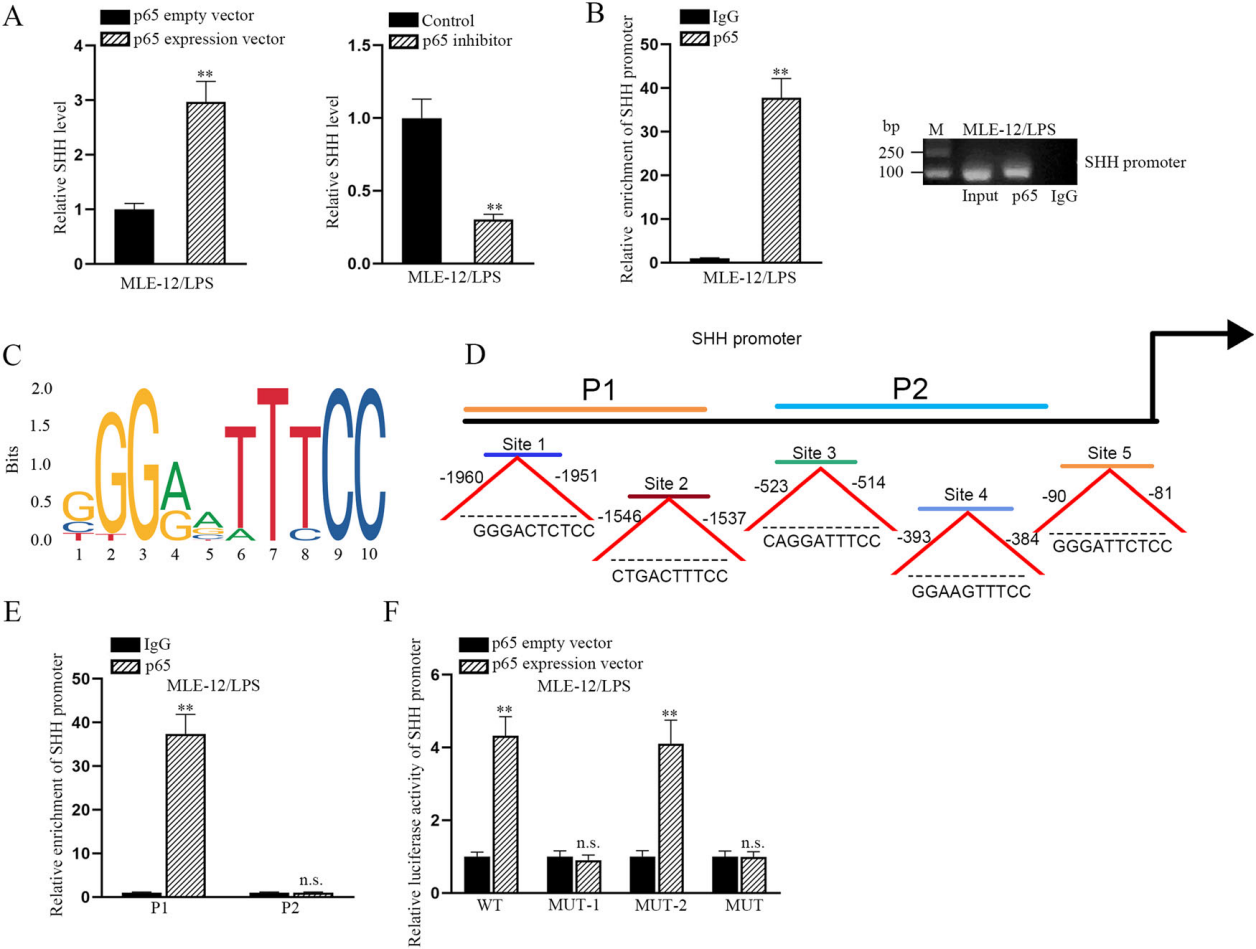
p65 facilitates the transcriptional activation of Shh in LPS-treated MLE-12 cells. **a** RT-qPCR detected the level of Shh in LPS-treated MLE-12 cells with p65 overexpression or inhibition. **b** ChIP assay examined the enrichment of Shh promoter induced by p65 or IgG in LPS-treated MLE-12 cell. **c**, **d** JASPAR predicted the DNA motif of p65 and its binding sites in Shh promoter. **e** ChIP assay detected the enrichment of Shh promoter on P1 or P2 in p65- or IgG-precipitated compounds in LPS-treated MLE-12 cells. **f** Luciferase reporter assays detected the luciferase activity of indicated Shh promoter (WT, MUT1, MUT2, and MUT) in LPS-treated MLE-12 cells under p65 overexpression or not. ^**^p < 0.01. n.s. no statistical significance.

Based on above data, we assumed that NF-κB pathway was responsible for the activation of hedgehog pathway. To demonstrate our hypothesis, we applied mechanism investigation to validate whether nuclear p65 activate the transcription of Shh, thus activating hedgehog pathway. The level of Shh mRNA was measured in LPS-treated MLE-12 cells transfected with p65 expression vector or p65 inhibitor. As expected, the mRNA level of Shh was positively regulated by p65 (Fig. 2a). ChIP assay proved the affinity of p65 to Shh promoter (Fig. 2b). The DNA motif of p65 and the binding sites for p65 in Shh promoter were obtained from JASPAR (http://jaspar.genereg.net/) (Fig. 2c, d). Further ChIP assay revealed that p65 had a high affinity in part 1 (P1) of Shh promoter (Fig. 2e). Based on the bioinformatics analysis, there were two binding sequences in P1 fragment. According to the result of luciferase reporter assay, site 1 was the functional site which was responsible for the binding of p65 to Shh promoter (Fig. 2f). Thus, we confirmed that nuclear p65 induces the transcriptional activation of Shh.

### MSC-secreted exosomes inactivate NF-κB pathway

**Fig. 3:**
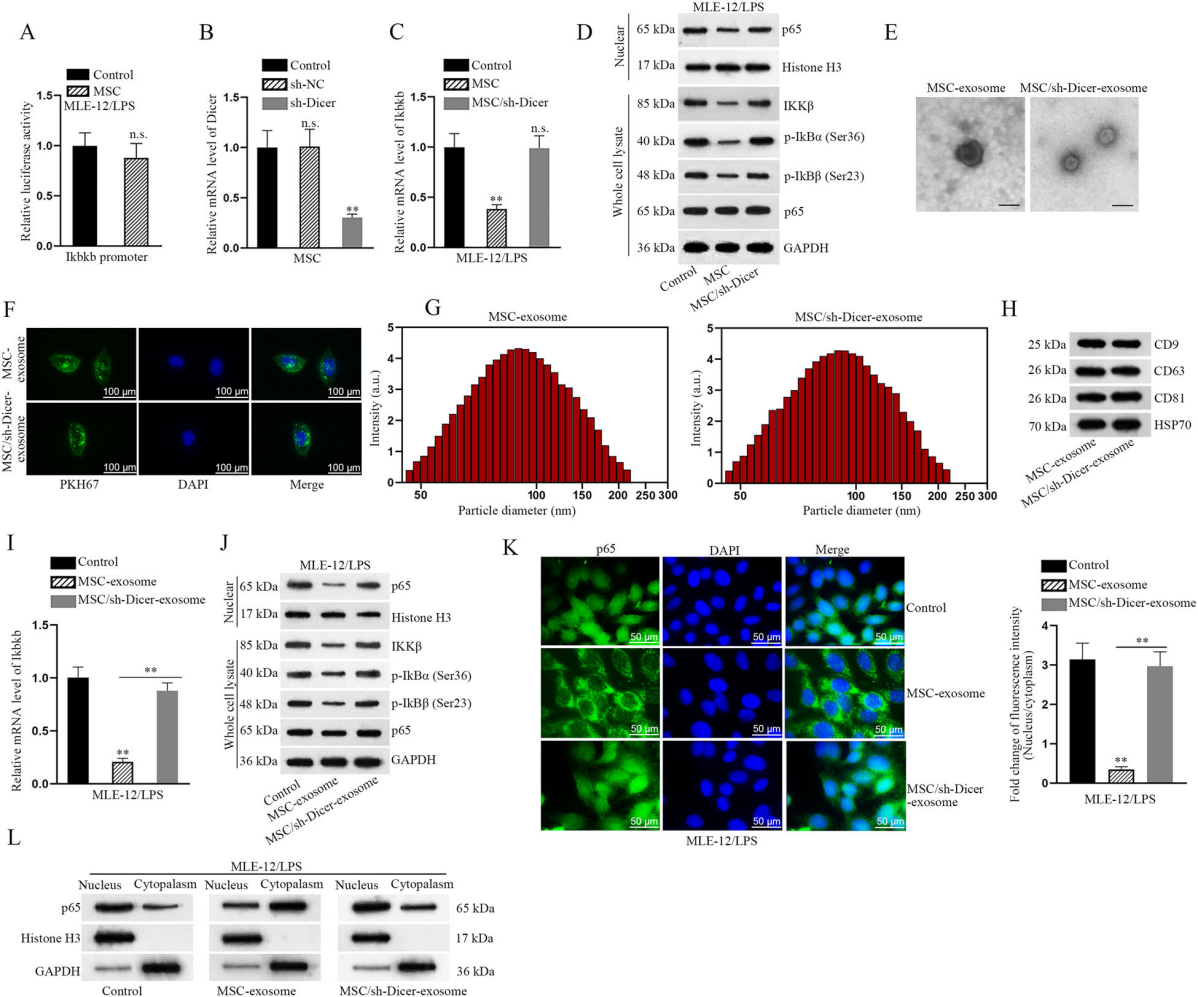
MSC-secreted exosomes inactivate NF-κB pathway. **a** Luciferase reporter assay detected the luciferase activity of Ikbkb promoter vector under coculture of MSC. **b**, **c** The relative mRNA level of Dicer and Ikbkb was examined by RT-qPCR in LPS-treated MLE-12 cells cocultured with MSC or Dicer-silenced MSC, respectively. **d** The protein levels of NF-κB pathway key factors (IKKβ, p-IκBα, and p-IKBβ in the whole cell lysate as well as the nuclear protein level of p65) were checked using western blot after cocultured with MSC or Dicer-silenced MSC. **e** Electron microscope analyzed the existence of exosomes secreted by MSC or Dicer-silenced MSC. **f** PKH67 staining was used to confirm the entrance of exosomes secreted by MSC or Dicer-silenced MSC into target cells. Scale bar = 100 μm. **g** The diameter of exosomes was measured and identified through NTA. **h** The surface markers of exosomes (CD9, CD63, CD81, and HSP70) were detected by western blot analysis. **i** RT-qPCR examined the level of Ikbkb mRNA in LPS-treated MLE-12 cells treated with MSC-exosome or MSC/sh-Dicer-exosome. **j** Western blot examined the protein levels of nuclear p65, Ikkβ, p-IκBα, and p-IκBβ in LPS-treated MLE-12 cells treated with MSC-exosome or MSC/sh-Dicer-exosome. **k** IF staining detected the nuclear translocation of p65 in LPS-treated MLE-12 cells under the context of MSC-exosome or MSC/sh-Dicer-exosome. Scale bar = 50 μm. **l** The cellular location of p65 was validated by western blot analysis in LPS-treated MLE-12 cells with MSC-exosome or MSC/sh-Dicer-exosome treatment. ^**^p < 0.01. n.s. no statistical significance.

We explored the regulatory mechanism by which Ikbkb mRNA level was changed. Based on the result of luciferase reporter assay, the luciferase activity of Ikbkb promoter vector was unchanged under the coculture of MSC (Fig. 3a). It is well-known that protein-coding genes are often modulated by microRNAs (miRNAs). Thus we suspected that miRNAs might involve in the modulation of Ikbkb. As we know, Dicer is a multi-domain protein which belongs to the RNase III family and plays a crucial role in processing precursor miRNAs (pre-miRNAs) into mature miRNAs^30^. To explore whether MSC-induced downregulation of Ikbkb through miRNAs, we silenced Dicer in MSC (Fig. 3b). Besides, we used Dicer-silenced MSC to coculture LPS-treated MLE-12 cells. Intriguingly, the mRNA level of Ikbkb was only impaired by MSC but not by MSC with silenced Dicer (Fig. 3c). Moreover, the protein levels of NF-κB pathway key factors were almost unchanged as well after cocultured with Dicer-silenced MSC (Fig. 3d). Based on these findings, we hypothesized that MSC might depend on miRNAs to downregulate Ikbkb in LPS-treated MLE-12 cells. Exosomes are crucial intercellular communicators, which can transfer RNA molecules from donor cells to recipient cells and therefore regulate the biological processes of recipient cells. In this study, we explored whether MSC secreted exosomes, thus transferring miRNAs into LPS-treated MLE-12 cells to regulate the apoptosis or EMT process of such cells. According to the experimental results of electron microscope, we identified the existence of exosomes secreted by MSC or Dicer-silenced MSC (Fig. 3e). Moreover, we applied PKH67 staining to further confirm that both the exosomes from MSC or Dicer-silenced MSC entered into LPS-treated MLE-12 cells (Fig. 3f). Subsequently, the diameter of exosomes was measured and identified through NTA, and the surface markers of exosomes (CD9, CD63, CD81, and HSP70) were verified by western blot. Results proved that no apparent differences were found between the exosomes from MSC with or without Dicer inhibition (Fig. 3g, h). To identify the role of exosomes in regulating Ikbkb-participated NF-κB pathway, we applied RT-qPCR and western blot analyses to examine the level of Ikbkb mRNA and that of NF-κB-related key proteins. The level of Ikbkb as well as the protein level of nuclear p65, IKKβ, p-IκBα, and p-IκBβ was significantly decreased in LPS-treated MLE-12 cells treated with MSC-exosome, whereas no significant changes of them were observed in LPS-treated MLE-12 cells with exosomes from Dicer-silenced MSC (Fig. 3i, j). The nuclear translocation of p65 was also detected in indicated LPS-treated MLE-12 cells. Compared with control group, the nuclear level of p65 was reduced in injured cells when treated with MSC-exosome, while was not changed in those treated with MSC/sh-Dicer-exosome (Fig. 3k). The location of p65 was further validated by western blot analysis. As shown in Fig. 3l, the level of nuclear p65 was reduced in MLE-12/LPS cells treated with MSC-exosome, whereas the abundance of p65 in nucleus was not changed in cells treated with exosomes from Dicer-silenced MSC cells. Functionally, we found that EMT process was attenuated in LPS-treated MLE-12 cells treated with exosome secreted by MSC but not Dicer-silenced MSC (Fig. S3A–C). Collectively, MSC-secreted exosomes transfer certain miRNAs targeting Ikbkb to inactivate NF-κB pathway in LPS-treated MLE-12 cells.

### MiR-182-5p transmitted by MSC-exosomes reverses EMT process by directly targeting Ikbkb

**Fig. 4:**
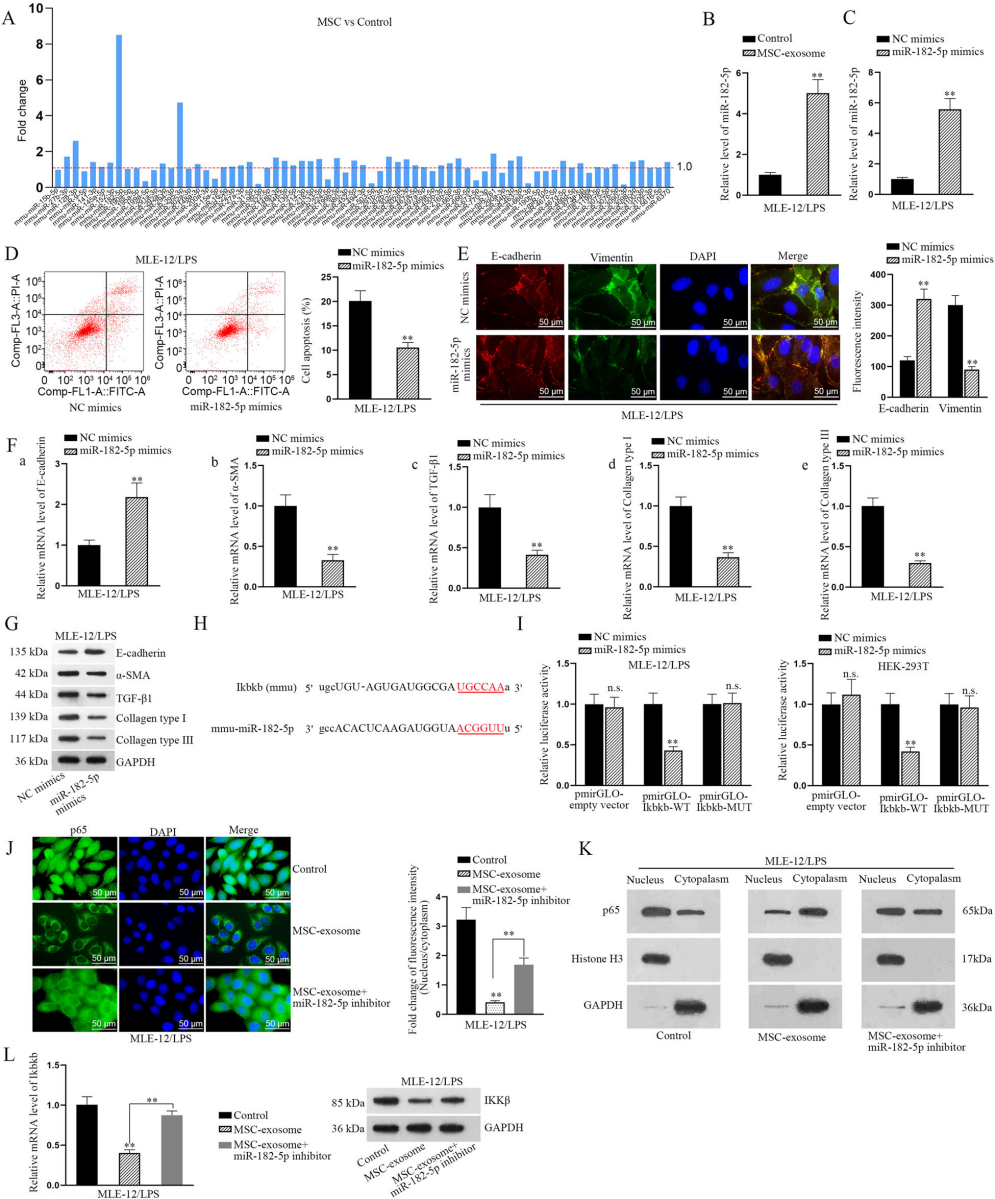
MiR-182-5p transmitted by MSC-exosomes reverses EMT process by directly targeting Ikbkb. **a** Online starBase v2.0 predicted 71 miRNAs targeted to Ikbkb were subjected to RT-qPCR analysis in LPS-treated MLE-12 cells under MSC coculture or not. **b** RT-qPCR analyzed the level of miR-182-5p in LPS-treated MLE-12 cells treated with MSC-exosome. **c** Relative level of miR-182-5p was detected by RT-qPCR in LPS-treated MLE-12 cells transfected with specific miRNA mimics. **d** Flow cytometry analysis displayed the apoptosis rate in LPS-treated MLE-12 cells transfected with miR-182-5p mimics. **e** IF assay analyzed the fluorescence intensities of two EMT-related proteins, E-cadherin, and Vimentin, in LPS-treated MLE-12 cells transfected with miR-182-5p mimics. Scale bar = 50 μm. **f**, **g** RT-qPCR and western blot examined the levels of epithelial marker (E-cadherin) and mesenchymal markers (α-SMA, TGF-β1, Collagen type I, and Collagen type III) in LPS-treated MLE-12 cells transfected with miR-182-5p mimics. **h** The binding sites between miR-182-5p and Ikbkb were predicted. **i** Luciferase reporter assay examined the luciferase activity of indicated reporter vectors in LPS-treated MLE-12 cells and HEK-293T cells co-transfected with miR-182-5p mimics or NC mimics. **j** IF assay detected p65 nuclear translocation in LPS-treated MLE-12 cells with MSC-exosome or MSC-exosome+miR-182-5p inhibitor. Scale bar = 50 μm. **k** Western blot analysis detected cytoplasmic and nuclear p65 in LPS-treated MLE-12 cells with MSC-exosome or MSC-exosome+miR-182-5p inhibitor. **l** RT-qPCR and western blot examined the mRNA and protein levels of Ikbkb in LPS-treated MLE-12 cells transfected with MSC-exosome or MSC-exosome+miR-182-5p inhibitor. ^**^p < 0.01. n.s. no statistical significance.

Next, we searched miRNAs that potentially targeted to Ikbkb from the online database starBase v2.0 (http://www.sysu.edu.cn/403.html). As a result, total of 71 candidate miRNAs with possibilities to bind to Ikbkb were screened out. Subsequently, these miRNAs were subjected to RT-qPCR analysis in MLE-12/LPS cells with or without MSC coculture. Interestingly, it manifested that among these 71 miRNAs, miR-182-5p was the most significantly upregulated one in MLE-12/LPS cocultured with MSC compared to that in those without (Fig. 4a). Further, the higher level of miR-182-5p was also detected in LPS-treated MLE-12 cells under the induction of MSC-exosome (Fig. 4b). To assess the biological role of miR-182-5p in LPS-treated MLE-12 cells, we overexpressed miR-182-5p with specific miRNA mimics (Fig. 4c). It was unveiled that LPS-induced cell apoptosis and EMT progress were both attenuated after overexpressing miR-182-5p (Fig. 4d–g). The binding sites between miR-182-5p and Ikbkb were predicted by starBase and illustrated in Fig. 4h. Luciferase reporter assay proved the interaction between miR-182-5p and Ikbkb (Fig. 4i). Furthermore, the results of IF staining suggested that the level of nuclear p65 weakened by MSC-exosome was partly recovered after inhibiting miR-182-5p expression (Fig. 4j). Meanwhile, the outcomes of western blot analyses also revealed that the level of p65 was reduced in nucleus with the treatment of MSC-exosome, while this tendency was partly reversed after co-treatment with miR-182-5p inhibitor (Fig. 4k). Intriguingly, we then disclosed that the decreased mRNA level of Ikbkb caused by MSC-exosome was totally rescued by miR-182-5p inhibitor, whereas the level of IKKβ protein impaired by MSC-exosome was partly recovered after miR-182-5p downregulation (Fig. 4l). Totally, miR-182-5p transmitted by MSC-exosomes reverses EMT process by directly targeting Ikbkb in LPS-treated MLE-12 cells.

### MSC-exosome induces the ubiquitination of IKKβ through downregulating Usp5

**Fig. 5:**
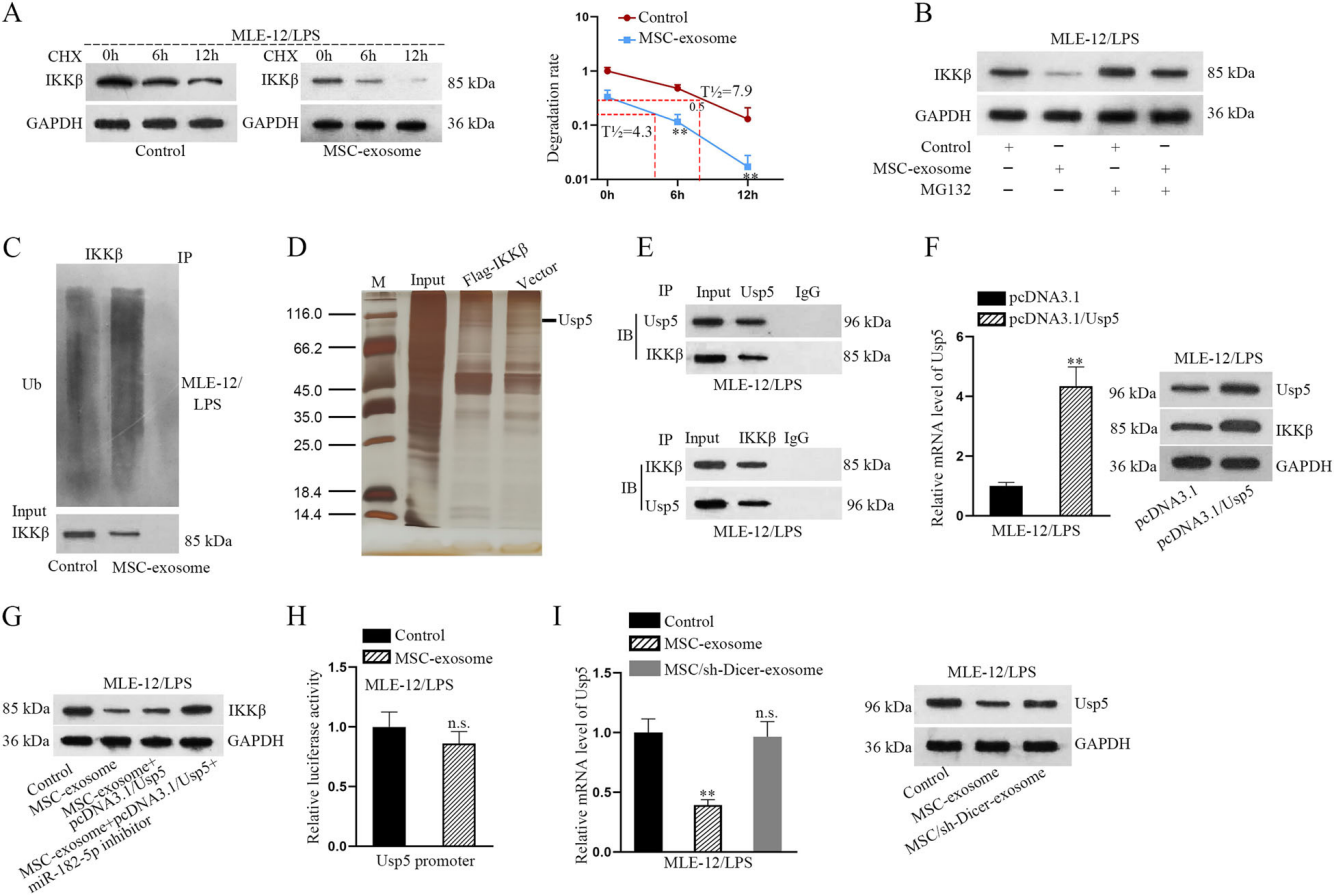
MSC-exosome induces the ubiquitination of IKKβ through downregulating Usp5. **a** After treated with CHX in three different time points, the protein level of IKKβ was tested in LPS-treated MLE-12 cells with or without MSC-exosome. **b** The protein level of IKKβ was detected in LPS-treated MLE-12 cells treated with control or MSC-exosome or control+MG132 or MSC-exosome+MG132. **c** Ubiquitination assay detected the ubiquitination of IKKβ protein in LPS-treated MLE-12 cells treated with MSC-exosome. **d** Pull-down silver staining was applied to unveil the proteins that might interact with IKKβ. **e** Co-IP assay demonstrated the interaction between Usp5 and IKKβ. **f** RT-qPCR and western blot examined the overexpression efficiency of Usp5 and the protein level of IKKβ in response to Usp5 overexpression. **g** Western blot analyzed the protein level of IKKβ in LPS-treated MLE-12 cells under four situations (control, MSC-exosome, MSC-exosome+pcDNA3.1/Usp5, or MSC-exosome+pcDNA3.1/Usp5+miR-182-5p inhibitor). **h** Luciferase reporter assays indicated the relative luciferase activity of Usp5 promoter in LPS-treated MLE-12 cells treated with MSC-exosome. **i** RT-qPCR and western blot detected the mRNA level and protein level of Usp5 in LPS-treated MLE-12 cells treated with MSC-exosome or MSC/sh-Dicer-exosome. ^**^p < 0.01. n.s. no statistical significance.

Based on above results, we further explored whether MSC-exosome regulated IKKβ protein at the post-translational level. After treated with CHX in three different time points, the protein level of IKKβ was tested in LPS-treated MLE-12 cells with or without MSC-exosome. Strikingly, the half-life of IKKβ protein was shortened after the treatment with MSC-exosome (Fig. 5a). Meanwhile, the protein level of IKKβ was found to be significantly decreased after treating with MSC-exosome but increased by the treatment with MG-132 (proteasome inhibitor). More importantly, the increased protein level of IKKβ caused by MG132 was not significantly changed after co-treatment with MSC-exosome (Fig. 5b). Ubiquitination assay revealed that MSC-exosome treatment strengthened the ubiquitination of IKKβ protein (Fig. 5c). Since numerous proteins can regulate the ubiquitination of functional proteins by protein–protein interaction, pull-down silver staining was applied to find certain ubiquitin-related proteins that may interact with IKKβ (Fig. 5d). In this case, a deubiquitinase ubiquitin specific peptidase 5 (Usp5) was chosen for further analysis. Co-IP assay further demonstrated the interaction between Usp5 and IKKβ (Fig. 5e). We then overexpressed Usp5 and found that the protein level of IKKβ was increased in response to Usp5 overexpression (Fig. 5f). We also evaluated whether Usp5 was involved in MSC-exosome-mediated IKKβ protein level. Data exhibited that the decreased level of IKKβ induced by MSC-exosome was partly rescued due to Usp5 overexpression, but was totally recovered after co-transfection with pcDNA3.1/Usp5 and miR-182-5p inhibitor (Fig. 5g). To examine whether MSC-exosome regulated Usp5 expression through affecting its transcription, we conducted luciferase reporter assays. Results indicated that MSC-exosome didn’t affect the transcriptional activity of Usp5 (Fig. 5h). Accordingly, we measured the mRNA and protein levels of Usp5 in LPS-treated MLE-12 cells with exosomes extracted from MSC or Dicer-silenced MSC. Both two levels of Usp5 were decreased after treatment with MSC-exosome while no significant change was observed in face of MSC/sh-Dicer-exosome (Fig. 5i), which implied that miRNAs were implicated in the modulatory process of MSC-exosome to Usp5. All these results suggested that MSC-exosome induces IKKβ ubiquitination through downregulating Usp5 in LPS-treated MLE-12 cells.

### MiR-23a-3p transmitted by MSC-exosome regulates the ubiquitination of IKKβ through targeting Usp5

**Fig. 6:**
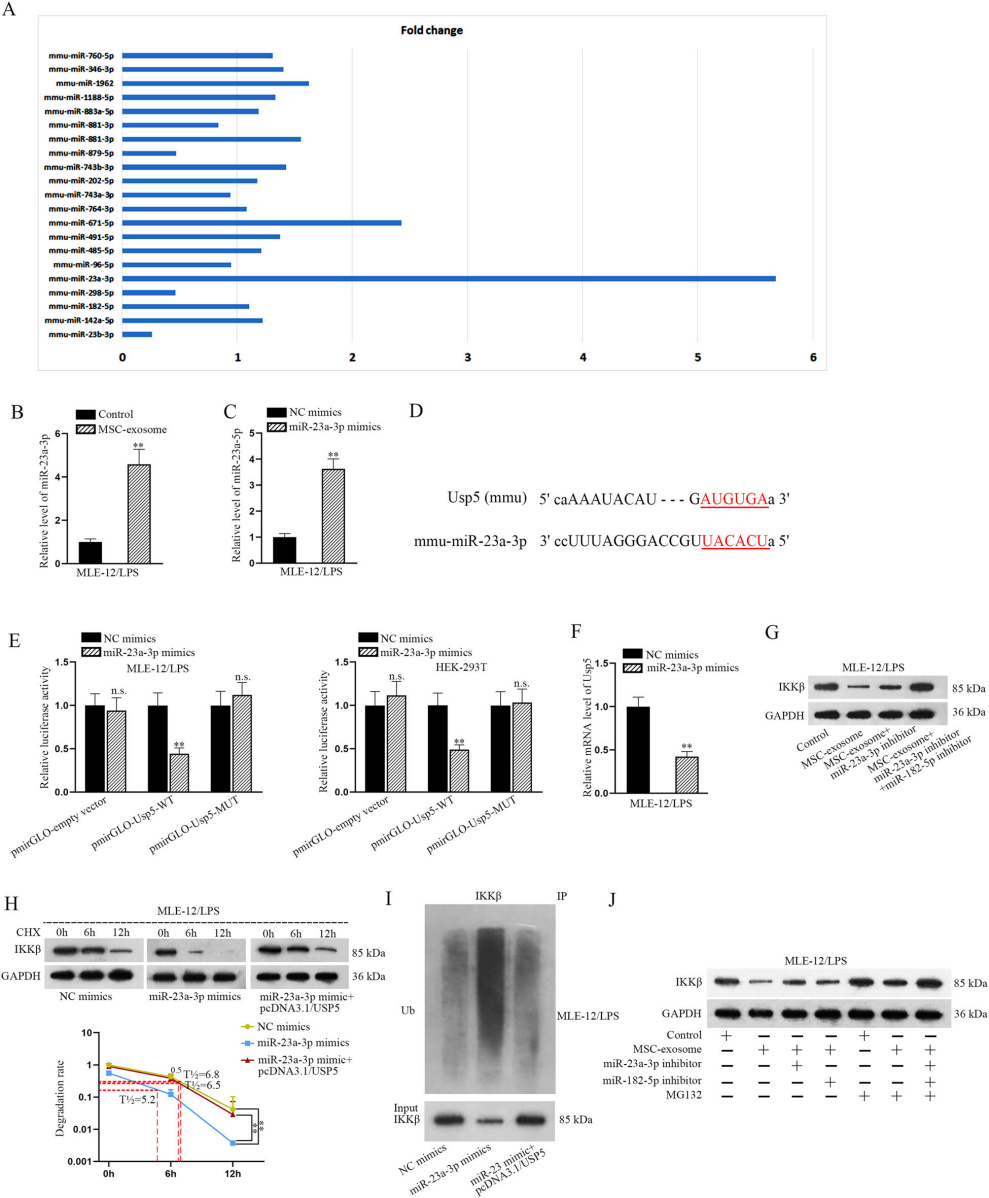
MiR-23a-3p transmitted by MSC-exosome regulates the ubiquitination of IKKβ through targeting Usp5. **a** StarBase v2.0 predicted miRNAs targeted to Usp5 were subjected to RT-qPCR analysis in LPS-treated MLE-12 cells with or without MSC coculture. **b** RT-qPCR analyzed miR-23a-3p expression in LPS-treated MLE-12 cells treated with MSC-exosome. **c** RT-qPCR analyzed miR-23a-3p expression in LPS-treated MLE-12 cells transfected with miR-23a-3p mimics. **d** The binding sequence between miR-23a-3p and Usp5 was shown. **e** Luciferase reporter assay examined the luciferase activity of indicated vectors in LPS-treated MLE-12 cells and HEK-293T cells co-transfected with miR-23a-3p mimics or NC mimics. **f** The expression level of Usp5 was detected by RT-qPCR in LPS-treated MLE-12 cells after the transfection of miR-23a-3p mimics. **g** Western blot measured the protein level of IKKβ in LPS-treated MLE-12 cells treated with different groups (control, MSC-exosome, MSC-exosome+miR-23a-3p inhibitor or MSC-exosome+miR-23a-3p inhibitor+miR-182-5p inhibitor). **h** After CHX treatment, the half-life of IKKβ was detected in LPS-treated MLE-12 cells transfected with NC mimics, miR-23a-3p mimics or miR-23a-3p mimics+pcDNA3.1/Usp5. **i** The ubiquitination level of IKKβ was detected in LPS-treated MLE-12 cells transfected with NC mimics, miR-23a-3p mimics or miR-23a-3p mimics+pcDNA3.1/Usp5. **j** Western blot examined the protein level of IKKβ in LPS-treated MLE-12 cells under seven conditions (control, MSC-exosome, MSC-exosome+miR-23a-3p inhibitor, MSC-exosome+miR-182-5p inhibitor, control+MG132, MSC-exosome+MG132, and MSC-exosome+miR-23a-3p inhibitor+miR-182-5p inhibitor+MG132). ^**^p < 0.01. n.s. no statistical significance.

According to above findings, we supposed that there might be a miRNA that were transferred by MSC-exosome to downregulate Usp5 in LPS-treated MLE-12 cells. Similarly, total 20 miRNAs possibly binding with Usp5 were predicted by analyzing starBase. To identify the one that could be released by MSC-exosome, we detected the expression of all these candidate miRNAs using RT-qPCR in MLE-12/LPS cells with or without MSC coculture. Of interest, among these 20 candidates, miR-23a-3p expression presented most marked enhancement in cells with MSC coculture relative to those without (Fig. 6a). In addition, elevated miR-23a-3p level was also tested in LPS-treated MLE-12 cells under the context of MSC-exosome (Fig. 6b). MiR-23a-3p was overexpressed for subsequent experiments (Fig. 6c). Functionally, upregulation of miR-23a-3p attenuated cell apoptosis and EMT progress induced by LPS (Fig. S4A–D). The binding sequence between miR-23a-3p and Usp5 was shown in Fig. 6d. The binding relation between them was further proven by luciferase reporter assay (Fig. 6e). The expression level of Usp5 was reduced after the transfection of miR-23a-3p mimics (Fig. 6f). Further, we discovered that the protein level of IKKβ reduced by MSC-exosome was partly recovered after miR-23a-3p inhibition, but was totally regained after co-suppression of miR-23a-3p and miR-182-5p (Fig. 6g). Subsequently, we investigated the role of miR-23a-3p/Usp5 axis in regulating IKKβ protein. As expected, the half-life of IKKβ was shorted in response to miR-23a-3p overexpression, whereas such phenomenon was reversed in response to Usp5 upregulation (Fig. 6h). In addition, the ubiquitination level of IKKβ was enhanced by upregulated miR-23a-3p but was attenuated after Usp5 overexpression (Fig. 6i). The protein level of IKKβ decreased by MSC-exosome was partly recovered after inhibition of miR-23a-3p or miR-182-5p alone. In MG-132-treated cells, we observed that the protein level of IKKβ was not significantly changed after treating with MSC-exosome or co-treating with MSC-exosome, miR-23a-3p inhibitor or miR-182-5p inhibitor (Fig. 6j). In short, miR-23a-3p transmitted by MSC-exosome contributes to the ubiquitination of IKKβ through targeting Usp5.

### Exosomal miR-23a-3p and miR-182-5p attenuates LPS-induced injury in MLE-12 cells by negatively regulating Usp5/Ikbkb axis

**Fig. 7:**
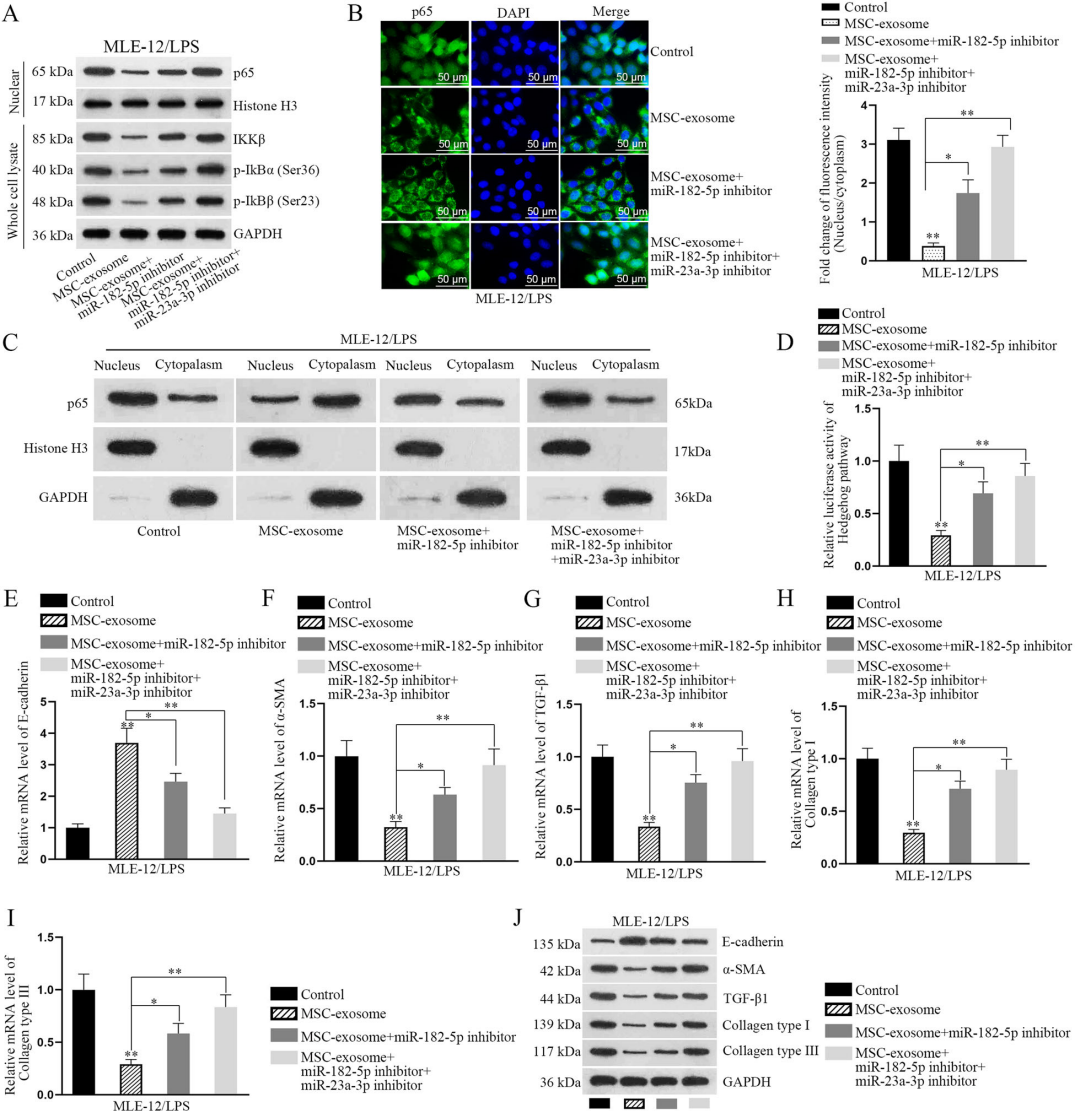
Exosomal miR-23a-3p and miR-182-5p attenuates LPS-induced injury and EMT process in MLE-12 cells by negatively regulating Usp5/Ikbkb axis. Rescue assays were carried out in LPS-treated MLE-12 cells under four different contexts (control, MSC-exosome, MSC-exosome+miR-182-5p inhibitor, and MSC-exosome+miR-182-5p inhibitor+miR-23a-3p inhibitor). **a** The levels of nuclear p65, IKKβ, p-IKBα, and p-IKBβ were detected in LPS-treated MLE-12 cells using western blot. **b** IF staining examined the nuclear translocation of p65 in LPS-treated MLE-12 cells under diverse conditions. Scale bar = 50 μm. **c** The protein level of p65 in nucleus or cytoplasm was examined by western blot analysis in LPS-treated MLE-12 cells with MSC-exosome, MSC-exosome+miR-182-5p inhibitor or MSC-exosome+miR-23a-3p inhibitor. **d** Luciferase reporter assay examined the luciferase activity of hedgehog pathway in LPS-treated MLE-12 cells. **e**–**i** RT-qPCR detected the mRNA level of E-cadherin, α-SMA, TGF-β1, Collagen type I, and Collagen type III in indicated LPS-treated MLE-12 cells. **j** Western blot detected the protein level of E-cadherin, α-SMA, TGF-β1, Collagen type I, and Collagen type III in indicated LPS-treated MLE-12 cells. ^*^p < 0.05, ^**^p < 0.01.

**Fig. 8:**
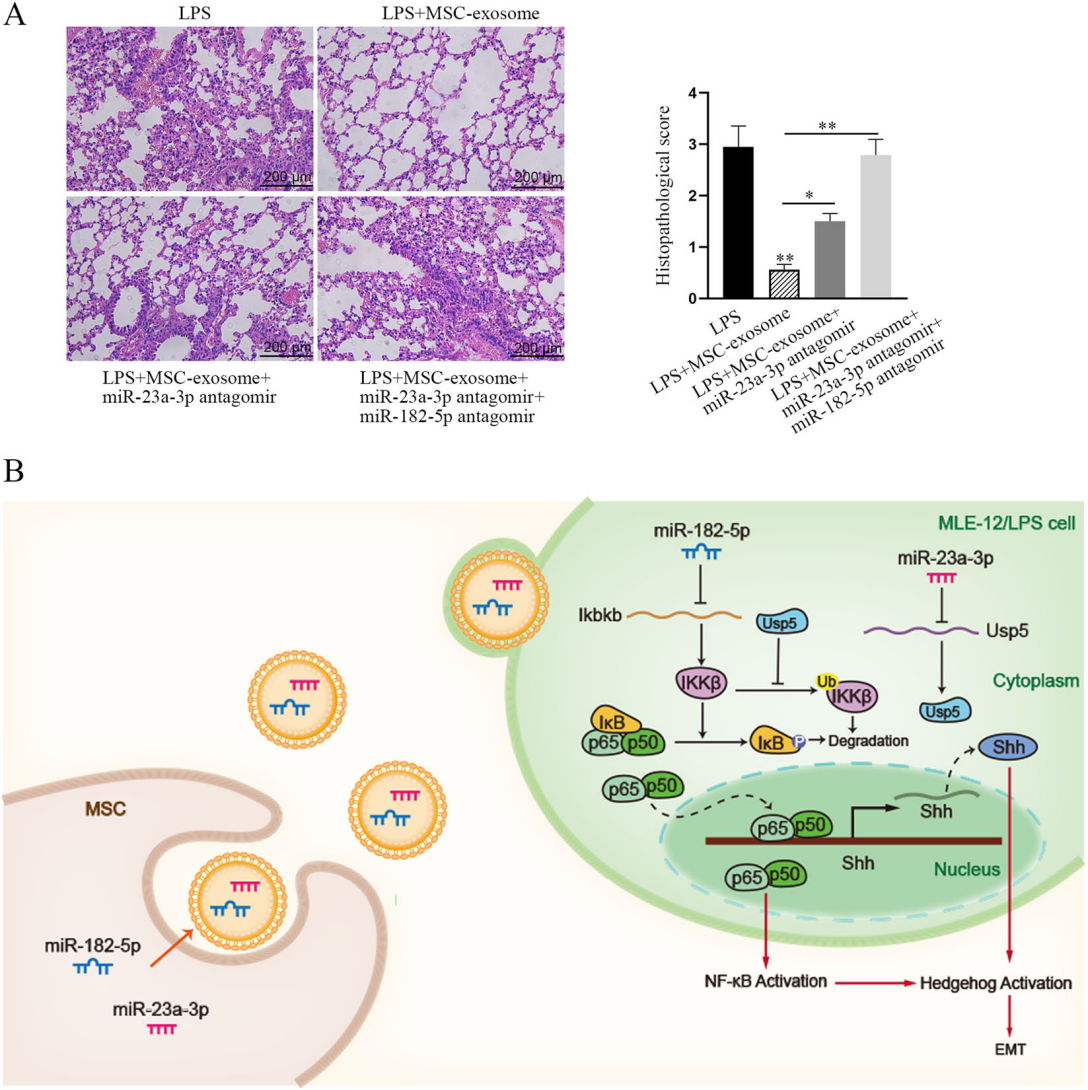
MSC-exosome delivered miR-182-5p and miR-23a-3p to mitigate LPS-induced ALI and pulmonary fibrosis by targeting Ikbkb/Usp5 signaling. **a** HE staining of lung tissues in four different groups (LPS, LPS + MSC-exosome, LPS + MSC-exosome+miR-23a-3p inhibitor, LPS + MSC-exosome+miR-23a-3p inhibitor+miR-182-5p inhibitor). Scale bar = 200 μm. **b** The concept map demonstrating the role and functional mechanism of MSC on alleviating ALI and pulmonary fibrosis. ^*^p < 0.05, ^**^p < 0.01.

Based on above data, we speculated that MSC-exosome impaired NF-κB pathway by delivering miR-23a-3p and miR-182-5p into LPS-treated MLE-12 cells. Here, such speculation was further proved by following phenomena. At first, we uncovered that the levels of nuclear p65, IKKβ, p-IKBα, and p-IKBβ decreased by MSC-exosome were partly recovered by miR-182-5p inhibitor, while was totally rescued by the co-inhibition of miR-182-5p and miR-23a-3p (Fig. 7a). Similar tendency was observed in the nuclear translocation of p65 (Fig. 7b, c). In addition, the activity of hedgehog pathway decreased by MSC-exosome was partially recovered by miR-182-5p inhibitor and was completely attenuated after co-inhibiting miR-23a-3p and miR-182-5p (Fig. 7d). Also, the expression of EMT markers was also measured under abovementioned conditions by RT-qPCR and western blot analyses. According to the results of Fig. 7e–j, we confirmed that the EMT process suppressed by MSC-exosome was partially recovered after miR-182-5p suppression but was completely recovered by the co-inhibition of miR-182-5p and miR-23a-3p. HE staining of the lung tissues showed that LPS-induced lung injury was alleviated by MSC-exosome, whereas the effect of MSC-exosome was partly abolished by the inhibition of miR-23a-3p and was totally abolished by the co-inhibition of miR-23a-3p and miR-182-5p (Fig. 8a). Accordingly, through analyzing the expression levels of EMT-related proteins in these lung tissues, we discovered that MSC-exosome mitigated EMT process in LPS-injured lung, and such mitigative effect could be partly offset by inhibited miR-23a-3p and almost completely counteracted by co-inhibition of miR-23a-3p and miR-182-5p (Fig. S5A). Of note, such phenomena seemed to be attributed to changes of key proteins involved in NF-κB and Hegdehog pathways in these lungs, which exhibited similar trends in response to the same conditions (Fig. S5B). In addition, we conducted rescue assays to determine the role of Usp5/Ikbkb axis in LPS-induced EMT process. Through measuring the levels of EMT markers, we determined that LPS-induced EMT progress was attenuated by MSC-exosome, whose impact was then partly recovered after Ikbkb upregulation but was completely rescued by overexpression of both Ikbkb and Usp5 (Fig. S6A, B). To support our above findings, we conducted some experiments with different conditions. It was uncovered that the mRNA level of Shh and the activity of hedgehog pathway were decreased after treating with MSC but were both increased by Ikbkb upregulation, and the decreased tendency caused by MSC was reversed by Ikbkb overexpression (Fig. S7A, B). These results indicated that NF-κB pathway contributed to the activation of hedgehog pathway. To examine whether LPS treatment affected the secretion of MSC-exosome, we examined the levels of exosomal markers (CD9, CD63, and CD81) in MSC exosome with or without treatment of LPS. Results showed that the levels exhibited no evident differences between above two conditions (Fig. S7C). Importantly, both the levels of miR-182-5p and miR-23a-3p were not changed in MSC-exosome when MSC was treated with or without LPS (Fig. S7D), excluding the effect of LPS on exosome secretion. Meanwhile, we unveiled that the expression levels of miR-182-5p and miR-23a-3p were decreased and even undetectable in the exosomes when Dicer was silenced in MSC (Fig. S7E). Collectively, our research findings indicated that MSC-exosome transmits miR-182-5p and miR-23a-3p into LPS-treated MLE-12 cells to respectively target Ikbkb and Usp5, thereby downregulating Ikbkb mRNA and destabilizing IKKβ protein to inactivate NF-κB and hedgehog pathways (Fig. 8b).

## Discussion

Mounting studies have revealed that MSC is helpful for ameliorating ALI and pulmonary fibrosis^14,15^. In line with previous reports, the present work discovered consistently that cell apoptosis and EMT process induced by LPS in MLE-12 cells can be partly alleviated by cocultured with MSC.

Based on the recent references, we learned that MSC reduces epithelial permeability following phosgene-induced ALI through regulating the classical signaling pathways, such as Wnt/β-catenin signaling pathway^31^ and NF-κB signaling pathway^32^. Here, we also investigated the effect of MSC on several classical signaling pathways in ALI cell model. We found that MSC could inactivate both NF-κB and hedgehog pathways. The crosstalk of signaling pathways can regulate disease development^33,34^. Here, we identified the activity of NF-κB and hedgehog pathways in the injured MLE-12 cells was impaired by MSC treatment. Intriguingly, previous study revealed the positive regulation of nuclear p65 on Shh^29^. Similarly, we confirmed that p65 was responsible for the transcriptional activation of Shh, thus activating hedgehog pathway. These findings prompted us to conclude that MSC reverses the injury and EMT process of LPS-treated MLE-12 cells through blocking NF-κB/hedgehog pathways.

In recent years, MSC has been proven to regulate disease progression by secreting exosomes^35–37^. Importantly, the role of MSC-exosome has been revealed in mitigating lung injury and fibrosis^38–40^. Here, we identified the role of MSC-exosome in relieving LPS-induced lung injury and EMT process. Intriguingly, our current study revealed that the levels of Ikbkb mRNA and IKKβ protein were both downregulated by MSC-exosome, while were not significantly changed by Dicer-silenced MSC-exosome. Excluding the transcriptional regulation of MSC-exosome on Ikbkb, we demonstrated that post-transcriptional modulation of MSC-exosome on Ikbkb. MiRNAs have been reported as the post-transcriptional regulators for mRNAs in various human diseases, including ALI^41,42^. MSC-exosomes have been reported as intercellular delivers to transfer miRNAs into ALI cells, thus regulating the functions of ALI cells. For examples, MSC-exosomes delivered miR-30b-3p inhibits SAA3 expression to protect against LPS-induced ALI^28^. Consistently, our study found that miR-182-5p transmitted by MSC-exosomes reversed EMT process by directly targeting Ikbkb in LPS-induced MLE-12 cells.

Post-translational modification is an important reason for the alteration of protein level^43–46^. Recently, ubiquitin-related proteins have been proven to be the regulators for the degradation of their target proteins through protein–protein interaction network. Considering the rescuing effect of miR-182-5p on MSC-exosome-mediated depletion of IKKβ was partial and incomplete, we further detected the mechanism responsible for the modification of IKKβ protein. Usp5 has been widely reported to be a stabilizer for proteins by de-ubiquitination^47–49^. Here, we confirmed the interaction between Usp5 and IKKβ and proved the effect of Usp5 on IKKβ stabilization. Combining with above data, we determined that Usp5 was targeted by miR-23a-3p that were transmitted by MSC-exosome. Hereto, we confirmed that exosomal miR-23a-3p secreted by MSC contributed to the ubiquitination of IKKβ through targeting Usp5. Importantly, we confirmed that LPS-treatment had no impact on the secretion of MSC-exosome, since the levels of exosome markers as well as miR-182-5p and miR-23a-3p levels were found to be not changed in MSC with or without LPS.

In conclusion, our work unveiled that MSC-exosome transmits miR-182-5p and miR-23a-3p into LPS-treated MLE-12 cells to respectively target Ikbkb and Usp5, thereby downregulating Ikbkb and destabilizing IKKβ to inactivate NF-κB and hedgehog pathways. All data revealed that MSC reverses the EMT process through blocking the activation of NF-κB and Hedgehog pathway in LPS-injured MLE-12 cells, which may provide utility value for the treatment of ALI and pulmonary fibrosis. Nonetheless, lack of clinical trial is a limitation of our current study, and great efforts remain to be made in our future study.

## Supplementary information

Supplementary figure legends

Figure S1

Figure S2

Figure S3

Figure S4

Figure S5

Figure S6

Figure S7

Supplementary file 1
