# Supplementary material for: Mesenchymal stem cells reverse EMT process through blocking the activation of NF-κB and Hedgehog pathways in LPS-induced acute lung injury
Source: Cell Death Dis. 2020 Oct 15;11(10):863. doi: 10.1038/s41419-020-03034-3 (PMC7567061; doi:10.1038/s41419-020-03034-3)
Supplement: Supplementary file 1 — Supplementary figure legends [file 41419_2020_3034_MOESM1_ESM.docx]

**Supplementary figure legends**

**Figure S1. LPS induces the apoptosis and EMT process in MLE-12 cells.** **A.** HE staining checked the injury of mice lung tissues in LPS group and the control group. Scale bar = 200μm. **B.** The apoptosis of MLE-12 cells treated with or without LPS was measured by flow cytometry analysis. **C.** EMT process was examined by IF staining in MLE-12 cells treated with or without LPS by testing the fluorescence intensities of E-cadherin and Vimentin. Scale bar = 50μm. **D-E.** The levels of epithelial marker (E-cadherin) and mesenchymal markers (α-SMA, TGF-β1, Collagen type I and Collagen type III) were assessed using RT-qPCR and western blot analyses. ^**^p < 0.01.

**Figure S2. MSC attenuates the apoptosis and EMT process in LPS-treated MLE-12 cells. A.** HE staining examined the lung injury induced by LPS or co-treatment with LPS+MSC. Scale bar = 200μm. **B.** Flow cytometry analysis revealed the apoptosis rate of LPS-treated MLE-12 cells with or without MSC co-culture. **C.** IF assay analyzed the fluorescence intensities of E-cadherin and Vimentin in LPS-treated MLE-12 cells co-cultured with MSCs. Scale bar = 50μm. **D-E.** RT-qPCR and western blot examined the levels of epithelial marker (E-cadherin) and mesenchymal markers (α-SMA, TGF-β1, Collagen type I and Collagen type III) in LPS-treated MLE-12 cells co-cultured with MSCs. ^**^p < 0.01.

**Figure S3. MSC-exosome can inhibit the EMT progress in** **LPS-treated MLE-12 cells. A.** IF assay analyzed the intensity of EMT markers (E-cadherin and Vimentin) in LPS-treated MLE-12 cells in following groups: control, MSC-exosome or MSC/sh-Dicer-exosome. Scale bar = 50μm. **B.** RT-qPCR detected the mRNA level of E-cadherin, α-SMA, TGF-β1, Collagen type I and Collagen type III in LPS-treated MLE-12 cells from indicated groups. **C.** Western blot detected the protein level of E-cadherin, α-SMA, TGF-β1, Collagen type I and Collagen type III in indicated LPS-treated MLE-12 cells. ^**^p < 0.01. n.s.: no statistical significance.

**Figure S4. MiR-23a-3p overexpression partly rescues the apoptosis and EMT process in LPS-treated MLE-12 cells. A.** Flow cytometry assessed the apoptosis of LPS-treated MLE-12 cells transfected with NC mimics or miR-23a-3p mimics. **B.** IF analyzed the intensity of EMT markers in LPS-treated MLE-12 cells with or without miR-23a-3p upregulation. Scale bar = 50μm. **C.** RT-qPCR detected the mRNA level of E-cadherin, α-SMA, TGF-β1, Collagen type I and Collagen type III in LPS-treated MLE-12 cells transfected with NC mimics or miR-23a-3p mimics. **D.** Western blot analyzed the protein levels of E-cadherin, α-SMA, TGF-β1, Collagen type I and Collagen type III in LPS-treated MLE-12 cells transfected with NC mimics or miR-23a-3p mimics. ^**^p < 0.01.

**Figure S5. MSC-exosome mitigates EMT in LPS-injured lung tissues by miR-23a-3p and miR-182-5p.**

**A**. Western blot detected the proteins related to EMT (including E-cadherin, α-SMA, TGF-β1, Collagen type I and Collagen type III) in the lung of mice treated with LPS, LPS+MSC-exosome, LPS+MSC-exosome+miR-23a-3p antagomir, or LPS+MSC-exosome+ miR-23a-3p antagomir+ miR-182-5p antagomir. **B**. The level of proteins associated with NF-κB and hedgehog pathways in above lungs was examined by western blot.

**Figure S6. MSC-exosome affects the EMT process through regulating Ikbkb and Usp5 expression in LPS-treated MLE-12 cells. A.** RT-qPCR detected the mRNA level of E-cadherin, α-SMA, TGF-β1, Collagen type I and Collagen type III in LPS-treated MLE-12 cells under the contexts of MSC-exosome, MSC-exosome+Ikbkb or MSC-exosome+Ikbkb+Usp5. **B.** Western blot analyzed the protein levels of E-cadherin, α-SMA, TGF-β1, Collagen type I and Collagen type III in LPS-treated MLE-12 cells under above conditions. ^*^P<0.05, ^**^p < 0.01.

**Figure S7. Identification of MSC-secreted exosome transmitted miR-182-5p and miR-23a-3p. A.** RT-qPCR analysis of Shh mRNA in LPS-treated MLE-12 cells under four different conditions (control, MSC, pcDNA3.1/Ikbkb and pcDNA3.1/Ikbkb+MSC). **B.** Relative luciferase activity of hedgehog pathway was measured in LPS-treated MLE-12 cells under above four conditions. **C.** The levels of exosome markers were examined in MSC-exosome treated with or without LPS were measured by western blot analysis. **D.** The levels of miR-182-5p and miR-23a-3p were detected by RT-qPCR in MSC-exosome when MSC was treated with or without LPS. **E.** The levels of miR-182-5p and miR-23a-3p in MSC-exosome derived from MSC transfected with or without sh-Dicer. ^**^p < 0.01, ^***^p < 0.001. n.s.: no statistical significance.
