# Supplementary figures and images for: Mesenchymal stem cells reverse EMT process through blocking the activation of NF-κB and Hedgehog pathways in LPS-induced acute lung injury
Source: Cell Death Dis. 2020 Oct 15;11(10):863. doi: 10.1038/s41419-020-03034-3 (PMC7567061; doi:10.1038/s41419-020-03034-3)

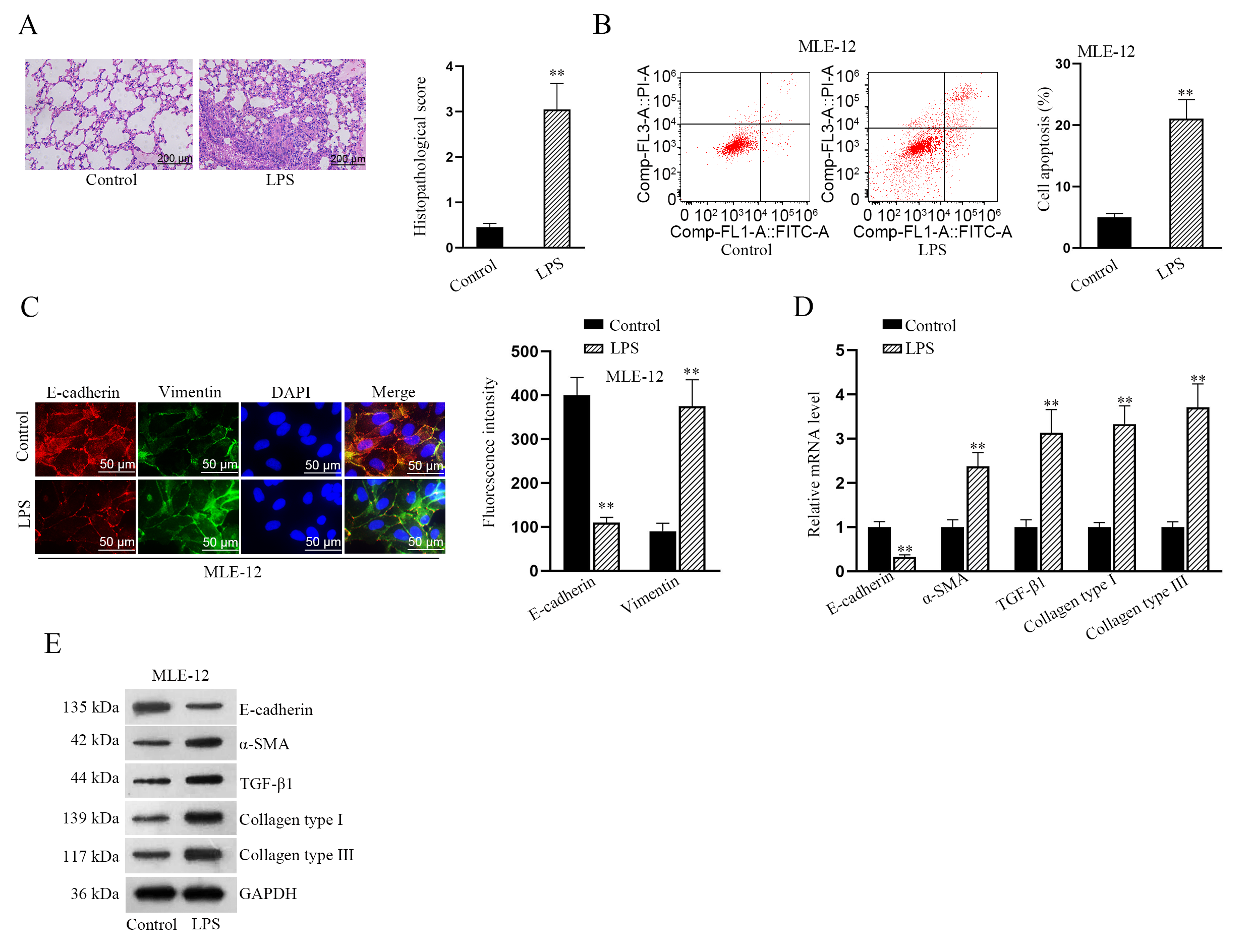

Supplement: Supplementary file 2 — Figure S1 [file 41419_2020_3034_MOESM2_ESM.tif]

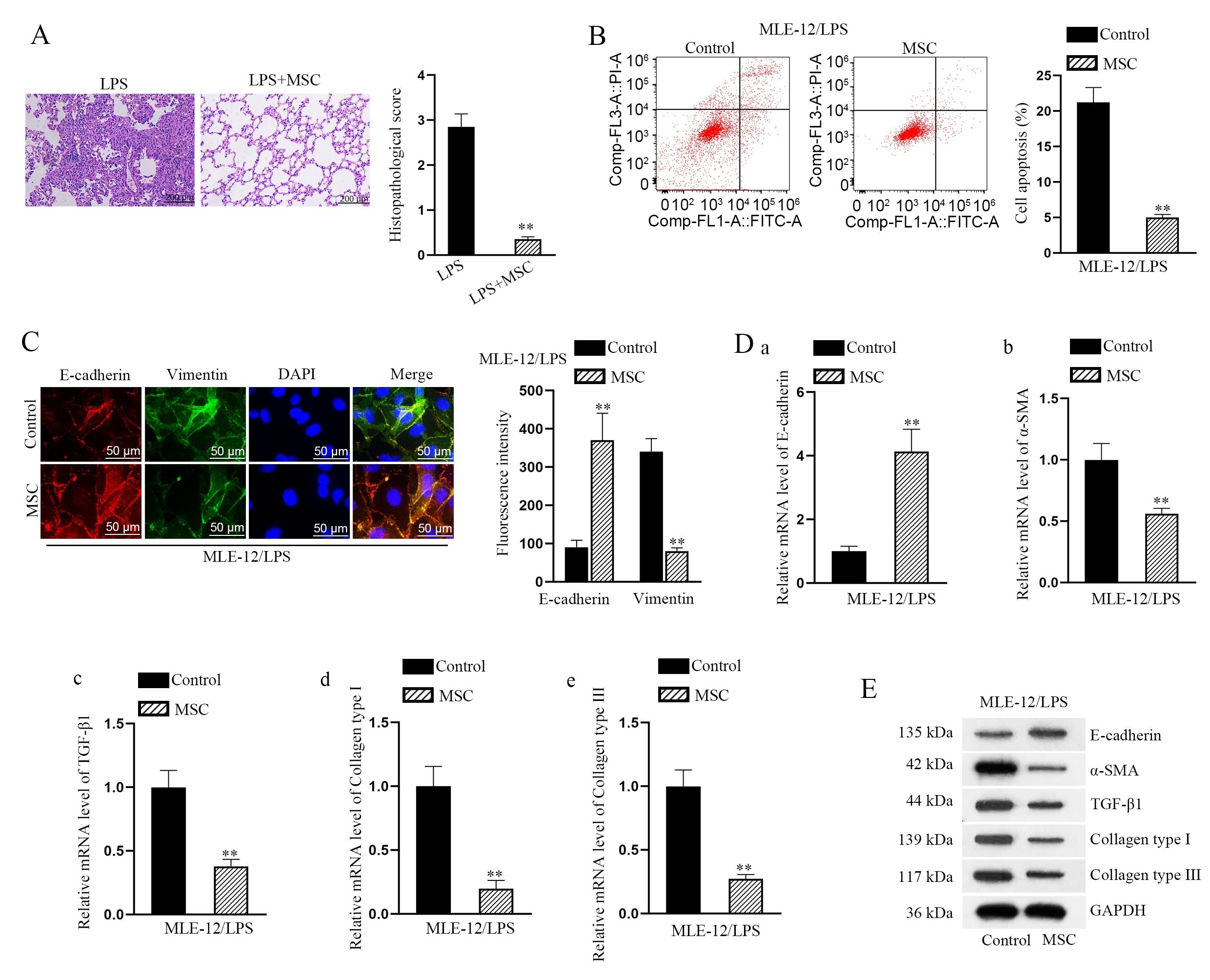

Supplement: Supplementary file 3 — Figure S2 [file 41419_2020_3034_MOESM3_ESM.tif]

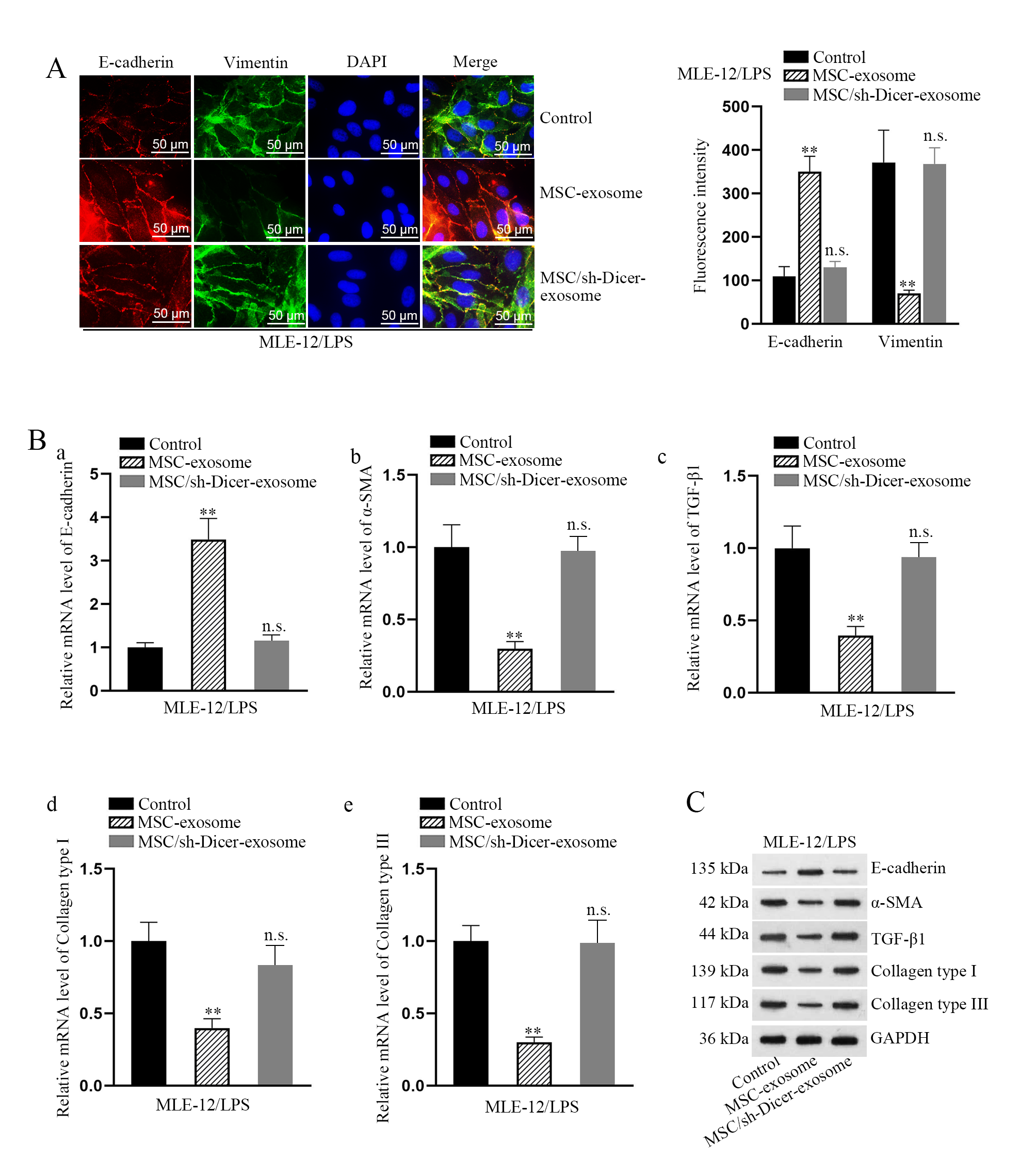

Supplement: Supplementary file 4 — Figure S3 [file 41419_2020_3034_MOESM4_ESM.tif]

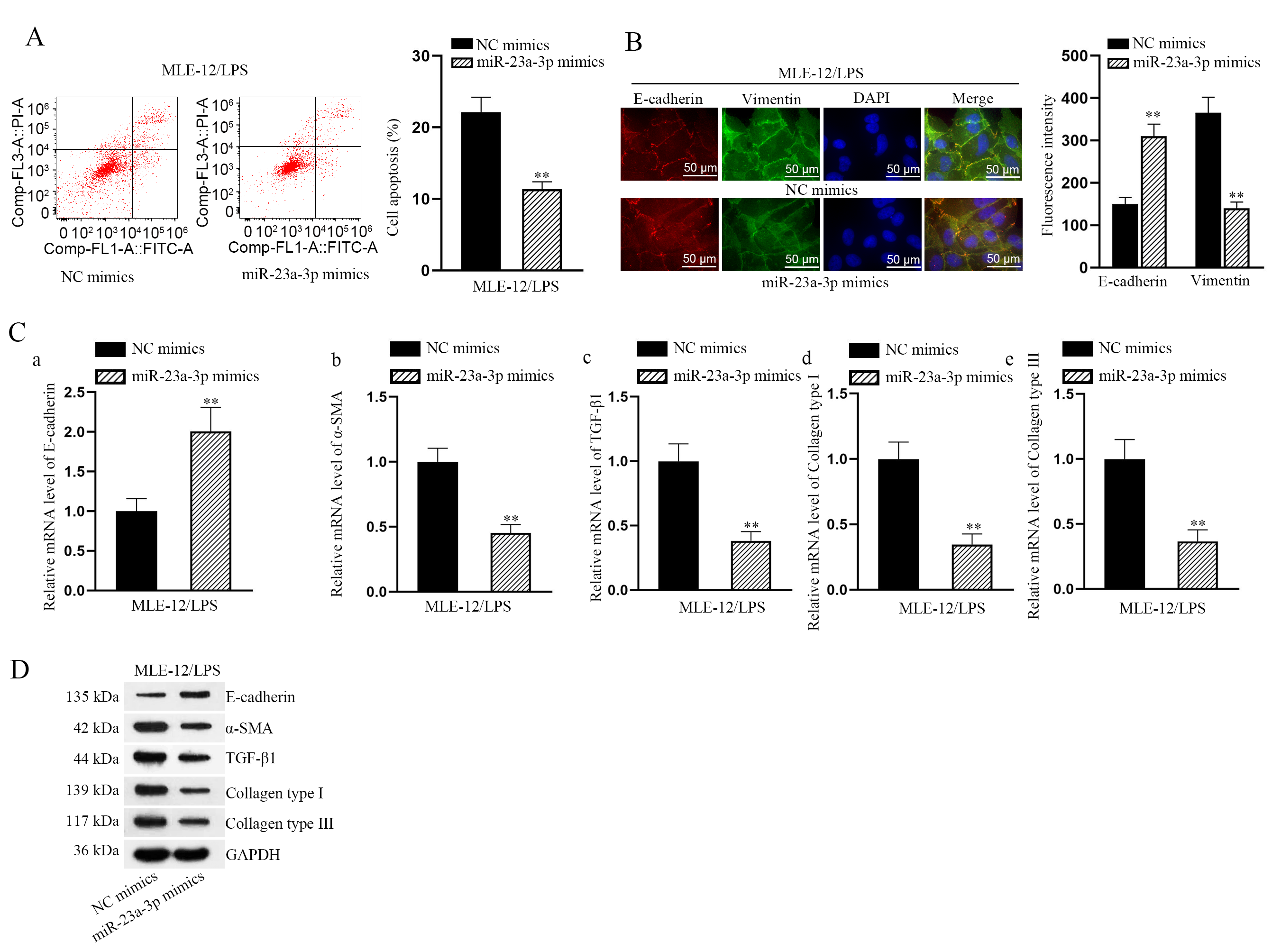

Supplement: Supplementary file 5 — Figure S4 [file 41419_2020_3034_MOESM5_ESM.tif]

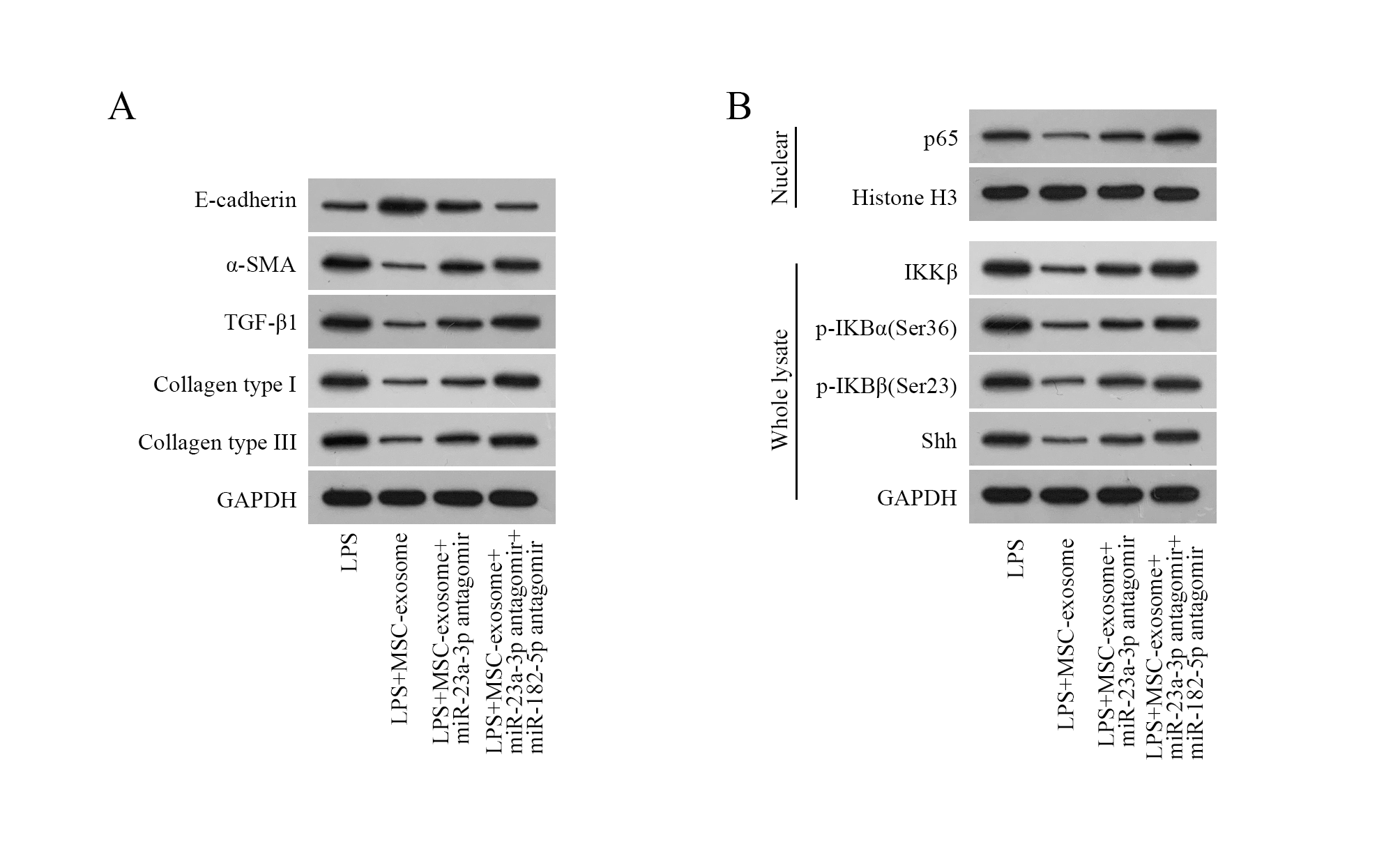

Supplement: Supplementary file 6 — Figure S5 [file 41419_2020_3034_MOESM6_ESM.tif]

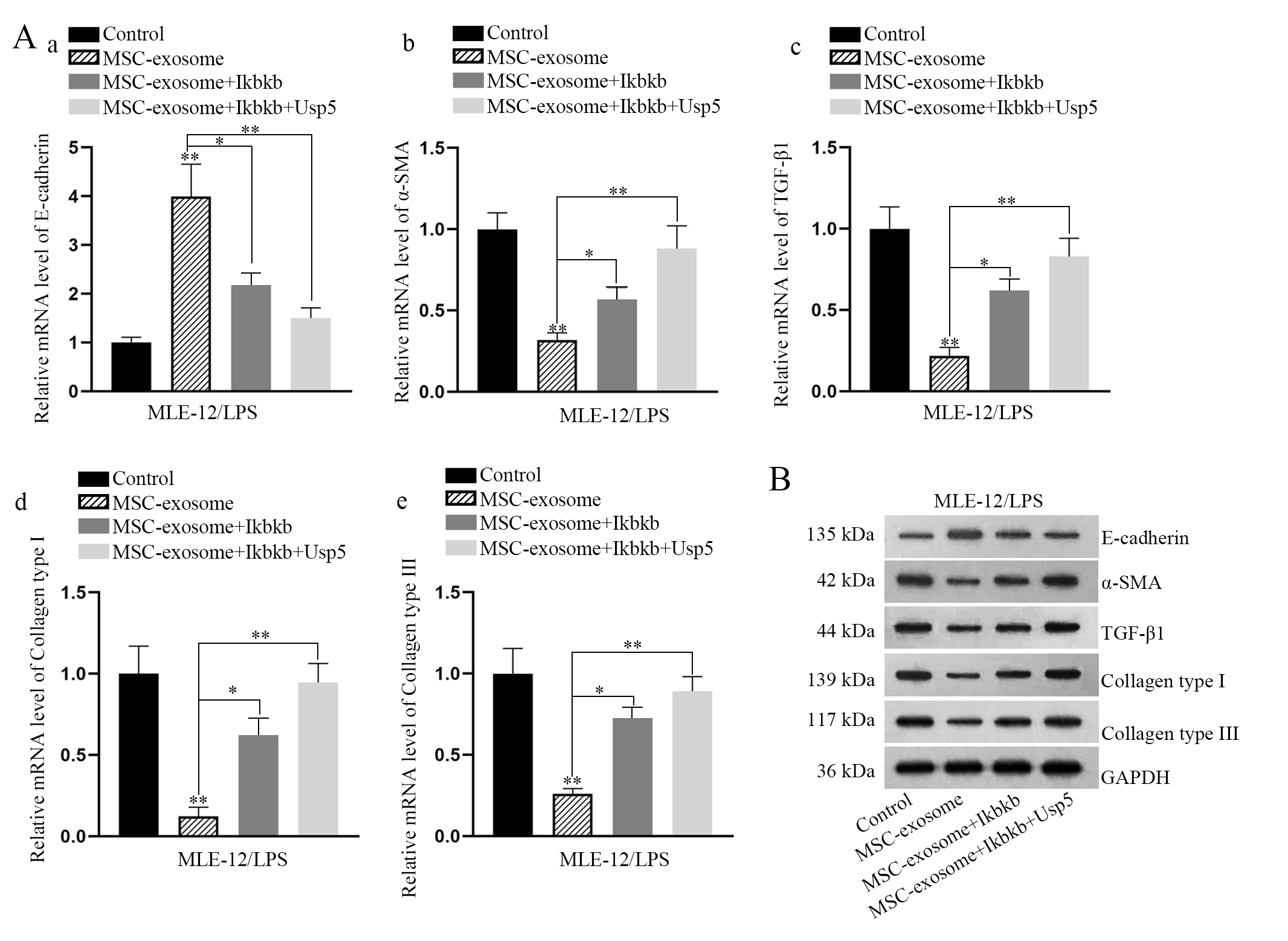

Supplement: Supplementary file 7 — Figure S6 [file 41419_2020_3034_MOESM7_ESM.tif]

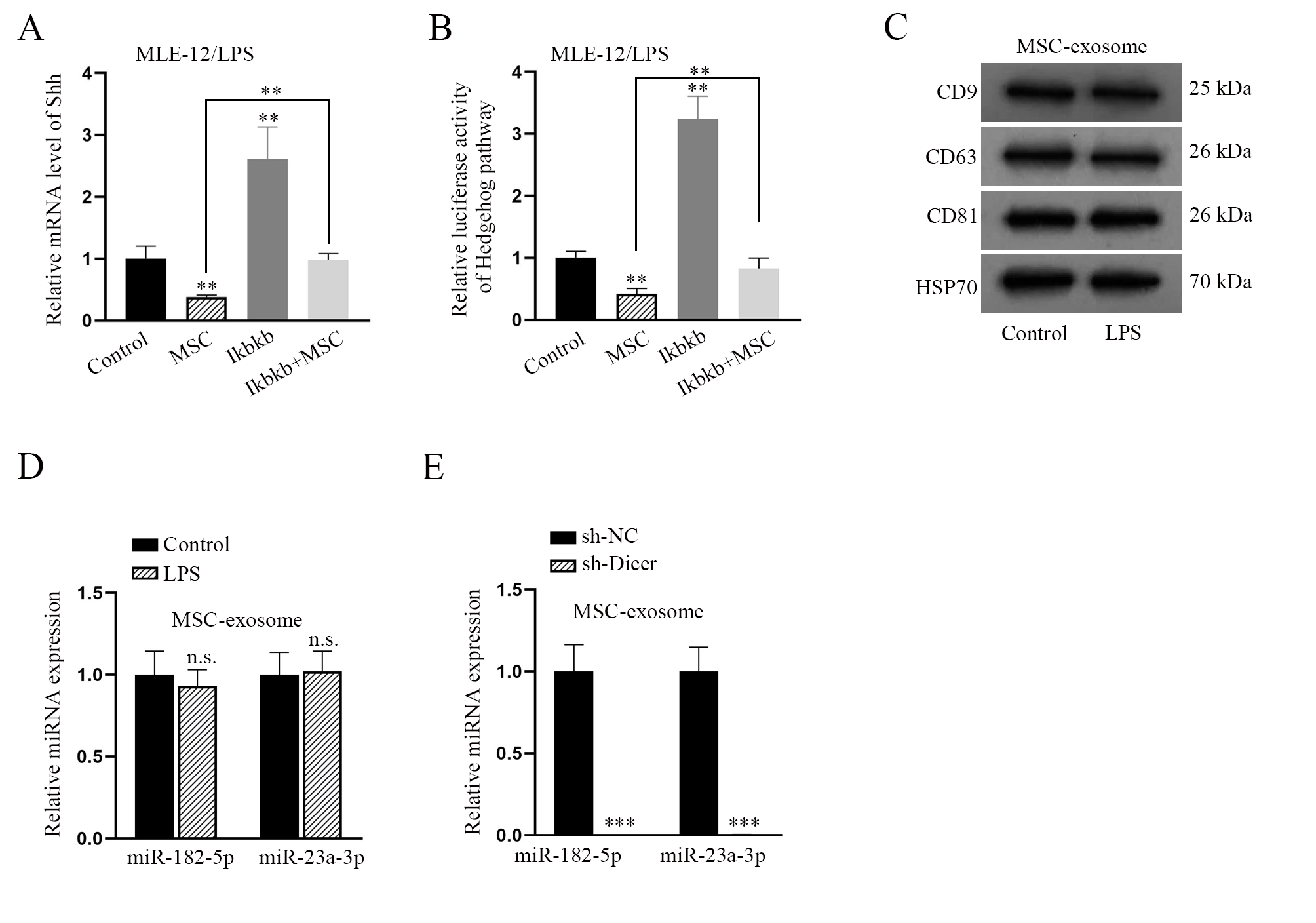

Supplement: Supplementary file 8 — Figure S7 [file 41419_2020_3034_MOESM8_ESM.tif]

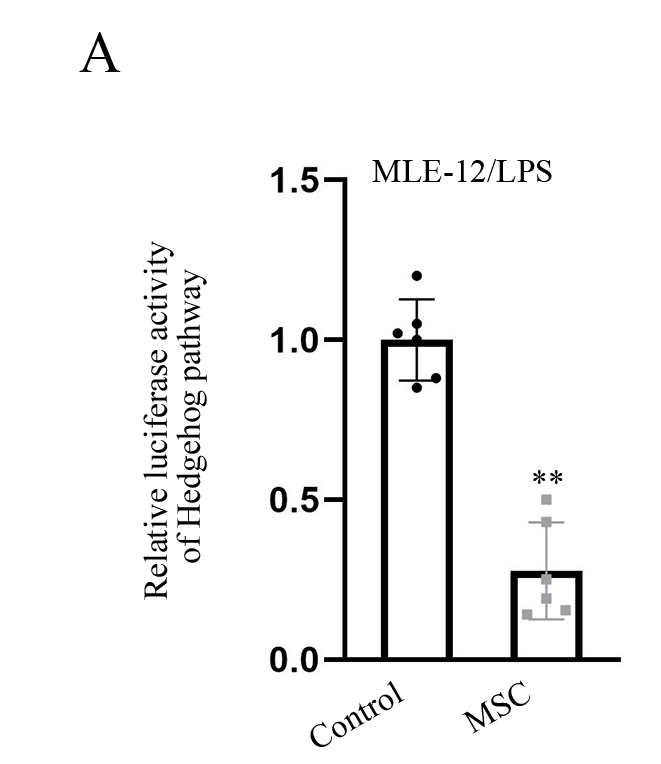

Supplement: Supplementary file 9 — Supplementary file 1 [file 41419_2020_3034_MOESM9_ESM.tif]
